# Stem cell‐derived extracellular vesicles reduce the expression of molecules involved in cardiac hypertrophy—In a model of human-induced pluripotent stem cell-derived cardiomyocytes

**DOI:** 10.3389/fphar.2022.1003684

**Published:** 2022-10-10

**Authors:** Alina Constantin, Ioana Karla Comarița, Nicoleta Alexandru, Alexandru Filippi, Florina Bojin, Mihaela Gherghiceanu, Alexandra Vîlcu, Miruna Nemecz, Loredan Stefan Niculescu, Virgil Păunescu, Adriana Georgescu

**Affiliations:** ^1^ Department of Pathophysiology and Pharmacology, Institute of Cellular Biology and Pathology “Nicolae Simionescu” of the Romanian Academy, Bucharest, Romania; ^2^ University of Medicine and Pharmacy “Carol Davila”, Bucharest, Romania; ^3^ Immuno-Physiology and Biotechnology Center (CIFBIOTECH), Department of Functional Sciences, “Victor Babes” University of Medicine and Pharmacy, Timisoara, Romania; ^4^ Center for Gene and Cellular Therapies in the Treatment of Cancer Timisoara-OncoGen, Clinical Emergency County Hospital “Pius Brinzeu” Timisoara, Timisoara, Romania; ^5^ “Victor Babeș” National Institute of Pathology, Bucharest, Romania

**Keywords:** extracellular vesicles (EVs), human-induced pluripotent stem cell-derived cardiomyocytes (hiPSC-CMs), human adipose tissue-derived stem cells (ADSCs), bone marrow-derived stem cells (BMMSCs), microRNAs (miRNAs), inflammation, cardiac hypertrophy

## Abstract

Cardiac pathological hypertrophy is the major risk factor that usually progresses to heart failure. We hypothesized that extracellular vesicles (EVs), known to act as important mediators in regulating physiological and pathological functions, could have the potential to reduce the cardiac hypertrophy and the ensuing cardiovascular diseases. Herein, the effects of mesenchymal stem cell-derived extracellular vesicles (EV-MSCs) on cardiac hypertrophy were investigated. EVs were isolated from the secretome of human adipose tissue-derived stem cells (EV-ADSCs) or bone marrow-derived stem cells (EV-BMMSCs). Human-induced pluripotent stem cell-derived cardiomyocytes (hiPSC-CMs) were stimulated with AngII and TGF-β1, in absence or presence of EVs. The results showed that exposure of hiPSC-CMs to AngII and TGF-β1 generated *in vitro* model of hypertrophic cardiomyocytes characterized by *increases* in *surface area*, reactive oxygen species production, protein expression of cardiac-specific biomarkers atrial natriuretic factor, migration inhibitory factor, cTnI, COL1A1, Cx43, α-SMA and signalling molecules SMAD2 and NF-kBp50. The presence of EV-ADSCs or EV-BMMSCs in the hiPSC-CM culture along with hypertrophic stimuli reduced the protein expressions of hypertrophic specific markers (ANF, MIF, cTnI, COL1A1) and the gene expressions of IL-6 molecule involved in inflammatory process associated with cardiac hypertrophy and transcription factors SMAD2, SMAD3, cJUN, cFOS with role in cardiomyocyte hypertrophic response induced by AngII and TGF-β1. The EV-ADSCs were more effective in reducing the protein expressions of hypertrophic and inflammatory markers, while EV-BMMSCs in reducing the gene expressions of transcription factors. Notably, neither EV-ADSCs nor EV-BMMSCs induced significant changes in cardiac biomarkers Cx43, α-SMA and fibronectin. These different effects of stem cell-derived EVs could be attributed to their miRNA content: some miRNAs (miR-126-3p, miR-222-3p, miR-30e-5p, miR-181b-5p, miR-124-3p, miR-155-5p, miR-210-3p hsa-miR-221-3p) were expressed in both types of EVs and others only in EV-ADSCs (miR-181a-5p, miR-185-5p, miR-21-5p) or in EV-BMMSCs (miR-143-3p, miR-146a-5p, miR-93-5p), some of these attenuating the cardiac hypertrophy while others enhance it. In conclusion, in hiPSC-CMs the stem cell-derived EVs through their cargo reduced the expression of hypertrophic specific markers and molecules involved in inflammatory process associated with cardiac hypertrophy. The data suggest the EV potential to act as therapeutic mediators to reduce cardiac hypertrophy and possibly the subsequent cardiovascular events.

## 1 Introduction

Hypertension is a major risk factor that contributes along with obesity, genetic predisposition and environmental factors to severity of cardiovascular disease, a leading cause of mortality worldwide. Chronically high blood pressure causes a hemodynamic overload and stimulates a cardiac hypertrophic response, that primarily is adaptive but, if the external or internal stimuli are persistent, a maladaptive and harmful response is initiated that affects heart function (Nadruz, 2015).

An early myocardial response is an increase in cardiac Angiotensin II (Ang II) protein levels, that *via* binding to its receptor, AT1-R, signals to induce cardiac hypertrophy or stimulates transforming growth factor-β1 (TGF-β1) expression in the myocardium, which also promotes hypertrophic responses in the heart (Takahashi et al., 1994; Fisher and Absher, 1995; Varagic and Frohlich, 2002). TGF-β1 first binds to the TGF-β type II receptor (TGF-β R2) at the cell surface stimulating it to activate the TGF-β type I receptor, which in turn phosphorylates downstream SMAD2 and SMAD3 proteins. Activated SMAD proteins translocate to the nucleus in complex with SMAD4, where they regulate the transcription of TGF-β target genes. A transcriptional program is activated and, cFOS and cJUN, components of AP-1, and their downstream target fetal genes including atrial natriuretic peptide (ANP) and B-type natriuretic peptide (BNP), are increased in cardiac hypertrophy. Enhanced protein synthesis in the absence of proliferation, the re-organization of the sarcomeres and extracellular matrix remodeling are some features of cardiomyocytes hypertrophy.

Human-induced pluripotent stem cells (hiPSCs) offer a valuable *in vitro* model for studying key molecular mechanisms involved in the cardiac diseases and testing new drugs safety and efficacy (Yu et al., 2007; Kadota et al., 2020). hiPSCs differentiated to cardiomyocytes (CMs) resemble human fetal rather than adult CMs in their structural, functional, and gene expression profiles (Gupta et al., 2010; van den Berg et al., 2015). Data from studies using hiPSCs improved the translation of the results to the clinical situation, and reduced the need for animal experiments, but there are still more questions that need answers (Giacomelli et al., 2020; Johansson et al., 2020).

The number of patients suffering of cardiovascular disease is increasing and thus, there is an urgent need for better treatment options. Current drug therapies improve most of the symptoms of patients with cardiovascular disease but with some of unwanted side effects. Cell transplants and construction of bio-artificial tissues are the primary strategies that have been performed, although in the last years have been published a lot of papers with studies where the effects of soluble factors have been investigated (Nicolas et al., 2017; Movileanu et al., 2021). Last advanced in biotechnology helped for the availability of human stem and progenitor cells with cardiac regenerative potential and this opened the door to regenerative medicine strategies for cardiac diseases (Liew et al., 2020). The mesenchymal stem cells (MSCs) capable of cardiac repair can be obtained from bone marrow, umbilical cord or peripheral blood and from adipose tissue. MSCs from adipose tissue (ADSCs) or bone marrow (BMMSCs), two of the most used MSC sources, have been shown to manifest different immunomodulatory properties and regenerative capacity (El-Badawy et al., 2016; Waldner et al., 2018). These features could be linked due to their paracrine activity and ability to secrete extracellular vesicles (EVs) (Constantin et al., 2020; Sid-Otmane et al., 2020). EVs are submicron vesicles released from a diversity of cell types that play an essential role in cell-to-cell communication by the release of intracellular content rich in lipids, proteins, mRNA and miRNAs that appear to change phenotypic and functional characteristics of the recipient cells (Nemecz et al., 2016; Alexandru et al., 2017). The circulating levels and properties of EVs are correlated with disease state and play a major role in atherosclerosis associated with inflammation, thrombosis as well as in CVD development and progression (Georgescu et al., 2009; Alexandru et al., 2019; Georgescu and Simionescu, 2021). Recent data have shown that although there is a high similarity between the most represented miRNAs in ADSC and BMMSC exosomes their relative proportions are different, thus these MSCs might deliver different information into their microenvironments (Baglio et al., 2015). Therefore, research on EVs produced by MSCs was intensified for use in cell-free regenerative medicine (Liew et al., 2020; Thompson et al., 2020).

In this study we investigated the therapeutic potential of EVs released by human MSCs isolated from adipose tissue (EV-ADSCs) or bone marrow (EV-BMMSCs) on human-induced pluripotent stem cell-derived cardiomyocytes (hiPSC-CMs) stimulated with Ang II and TGF-β1 as an *in vitro* model for cardiac hypertrophy. We reported here that both types of vesicles, EV-ADSCs and EV-BMMSCs, significantly decreased hypertrophic specific markers and molecules involved in inflammatory process associated with cardiac hypertrophy, and these effects could be partly due to their content in microRNA (miRNA). Our findings suggest that stem cell free derivatives-based models may provide new alternatives for therapeutic strategies targeting cardiovascular disease.

## 2 Materials and methods

### 2.1 Cardiomyocyte culture: Cellular model of hypertrophic cardiomyocytes

Nowadays, human-induced pluripotent stem cells (hiPSCs) represent a powerful cell-based system for *in vitro* differentiation of adult progenitor cells into cardiomyocytes to obtain information about mechanisms involved in the cardiovascular diseases that cannot be obtained by using animal experimental models.

Human cardiomyocytes (Pluricyte Cardiomyocytes) used in this study were ventricular cardiomyocytes derived from hiPSC, and were provided by Ncardia (Gosselies, Belgium). Thawing and culturing of human-induced pluripotent stem cell-derived cardiomyocytes (hiPSC-CMs) were carried out according to the manufacturer’s protocols. In short, after thawing and calculating cell viability by trypan blue exclusion using a hemocytometer, the hiPSC-CMs were cultured on culture plates coated with fibronectin in PBS (phosphate buffered saline) with Ca^2+^/Mg^2+^ (final concentration 10 μg/ml) (Sigma-Aldrich, Cat. No. F1141) in Pluricyte^®^ Cardiomyocyte medium (PC medium), a serum-free, chemically defined medium designed to promote cardiomyocyte maturation, and maintained at 37°C in a humidified incubator with 5% CO_2_ for 8 days. PC medium was refreshed starting with the first day after the hiPSC-CMs have cultured and then every 2 days until the eighth day, when the cells attained a mature phenotype and could be used in subsequent experiments. It is worth mentioning that the spontaneously beating phenotype of the hiPSC-CMs was visualized under a phase-contrast microscope, starting with the second day in culture.

To create an *in vitro* model of cardiac hypertrophy, hiPSC-CMs were stimulated with 200 nM AngII (Leenders et al., 2010), 10ng/ml TGF-β1 (Leenders et al., 2010; Watkins et al., 2012), or with a combination of both, AngII and TGF-β1, for 48 h.

#### 2.1.1 The quantitation of surface area of cardiomyocytes

To prove the hypertrophic phenotype of hiPSC-CMs thus stimulated, these were stained with FITC-labelled wheat germ agglutinin (WGA), a lectin that binds glycoproteins on the surface of live or fixed cells and marks fibrotic tissue. Also, DAPI (4′, 6-diamidino-2-phenylindole) staining was used to determine the number of nuclei. An image processing program, ImageJ 1.48v Macro, was used to count hiPSC-CMs (as indicated by DAPI-stained nuclei) and measure cell surface area (by WGA-stained membranes). Specifically, the user selected the leaflet area from bright-field images and reported the ratio between the cell surface area and number of nuclei. The surface area in control cells (unstimulated hiPSC-CMs) was normalized as 100%, and data from stimulated hiPSC-CMs were expressed as the fold change in cell size compared with the level measured in control cells.

#### 2.1.2 Real-time cell proliferation assay of cardiomyocytes

To assess the hypertrophic growth of cardiomyocytes we used a real time cell analyzer system**,** xCELLigence Real-Time Cell Analyzer (RTCA DP version; Roche, Mannheim, Germany), which provides a quantitative readout of cell number and proliferation rate by recording the impedance changes upon cell attachment or detachment from the surface electrodes.

In brief, 50 µL of fibronectin coating solution (10 μg/ml in PBS containing Ca^2+^ and Mg^2+^) was added to each well of the E-plate^®^ and incubated overnight at 4°C. The following day, excess fibronectin was aspirated, 180 µL of pre-warmed PC medium was added to each well and the plate was equilibrated in a 5%CO2 incubator at 37°C. After 30 mins, the plate was transferred to the xCELLigence^®^ RTCA DP in the incubator and background impedance measured for 3 min as per the manufacturer’s protocol. Next, the hiPSC-CMs were seeded in E-plate at a density of 3×10^4^ cells/well in PC medium under the following experimental conditions: the unstimulated hiPSC-CMs and hiPSC-CMs stimulated with 200 nM AngII, or 10 ng/ml TGF-β1, or with 200 nM AngII and 10 ng/ml TGF-β1. The plate was left in the laminar flow hood for 30 min to allow the cells to settle and attach after which it was transferred to the xCELLigence^®^ RTCA DP in the incubator. Impedance readings were automatically recorded every 15 min. At 24 h, the instrument was paused, the plate transferred to the laminar flow hood in a transfer module and the PC medium was replaced with a pre-warmed PC medium containing 10% FBS (fetal bovine serum). Again, the E-plate was locked in the RTCA DP device, and the cells were monitored every 15 min for up to 90 h; the experiments were run in duplicate. The impedance values were acquired automatically with the RTCA software (version 1.2.1) and expressed as Cell Index values (CI).

#### 2.1.3 Protein expression assessment by western blot analysis

The hiPSC-CMs cultured under the above experimental conditions, unstimulated or stimulated for 48 h with 200 nM AngII and 10 ng/ml TGF-β, were lysed in RIPA buffer with protease and phosphatase inhibitors (ThermoFisher Scientific). Proteins were separated on 10% SDS-PAGE on denaturing conditions and transferred to a nitrocellulose membrane. After blocking with 5% milk in 0.1%, PBS-Tween the membrane was incubated with primary antibodies overnight at 4°C. The following antibodies were used: rabbit polyclonal anti-phospho SMAD2/SMAD3 (ThermoFisher Scienific), rabbit polyclonal anti-SMAD2/SMAD3 (ThermoFisher Scienific), rabbit polyclonal anti-p65 NF-kB antibody (1:200 dilution, Santa Cruz Biotehnology), rabbit polyclonal anti-p50NF-kB (1:200 dilution, Santa Cruz Biotehnology) and rabbit anti-GAPDH (#ab9485, dilution 1:2000, Abcam). Subsequently, the membrane was washed and incubated with appropriate horseradish peroxidase-conjugated secondary antibody (goat anti-rabbit, 1:5000 dilution; Life Tehnologies). Immunoreactive protein bands were developed with an enhanced chemiluminescence kit (Amersham Pharmacia Biotech, United Kingdom Ltd., Little Chalfont, Buckinghamshire, United Kingdom) and developed on ImageQuant LAS 4000 (GE Healthcare, Chicago, United States). Bands intensities were quantified with TotalLab Quant software, and the expression level of each protein was normalized to GAPDH.

#### 2.1.4 Quantification of intracellular reactive oxygen species in cardiomyocytes

Generation of intracellular reactive oxygen species (ROS) was ascertained using 2′,-7′-dichlorofluorescein diacetate (DCFH-DA, Sigma-Aldrich) that undergoes two-electron oxidation in the presence of ROS to yield a fluorescent compound, 2′,7′-dichlorofluorescein (DCF). The unstimulated hiPSC-CMs or hiPSC-CMs stimulated for 48 h with 200 nM AngII and 10 ng/ml TGF-β1 were seeded in 96-well black plates at a density of 3–10^4^ cells/well, starved by serum deprivation for 24 h, and incubated for the next 48 h as described above. Then, medium was changed to HBSS buffer containing 10 μM DCFH-DA, and both unstimulated and stimulated hiPSC-CMs were kept in the dark for 1 h at 37°C. After incubation, the cells were washed with PBS and imaged by fluorescence microscopy (Axio Vert, Zeiss) (Nemecz et al., 2018). Three fluorescent images were taken for each condition and at least 50–100 cells were imaged at one time for each condition. DCF fluorescence intensity in control cells (unstimulated hiPSC-CMs) was considered 100% (control), and results were expressed as mean percentages relative to control ±standard deviation (SD).

### 2.2 Human mesenchymal stem cells

This study was conducted in accordance with the Declaration of Helsinki, and approved by Ethics Committees of the Institute of Cellular Biology and Pathology „Nicolae Simionescu” and Center for Gene and Cellular Therapies in the Treatment of Cancer Timisoara-OncoGen, Clinical Emergency County Hospital “Pius Brinzeu” Timisoara. Informed consent was obtained from all subjects involved in the study.

#### 2.2.1 Isolation and expansion of human adipose-derived stem cells

Human lipoaspirates of healthy normal weight subjects were collected during lipoplasty procedure after the informed consent filled up and signed by the donors. The protocol for the isolation of human adipose-derived stem cells (ADSCs) was presented in a previous paper published by our group (Constantin et al., 2017). Briefly, the lipoaspirate was digested with 0.075% collagenase type II in HBSS (Hanks’ balanced salt solution), and after filtration and centrifugation, the cells from the pellet known as adipose stromal vascular fraction (SVF) cells were plated in DMEM/F-12 (Dulbecco’s Modified Eagle Medium/Nutrient Mixture F-12) supplemented with 10% FBS (v/v), 2 mM glutamine, and antibiotics (100units/ml penicillin, 100 μg/ml streptomycin, 50 μg/ml neomycin) at 37°C in a humidified atmosphere containing 5% CO_2_. When the cells reached about 80–90% confluency (passage 0), these were detached with trypsin-EDTA buffer, and seeded at a density of 5000 cells/cm2 (passage 1). After two to 3 days, when the cells reached optimal confluency (80–90%), they were trypsinized and collected for further passages.

#### 2.2.2 Isolation and expansion of human bone marrow-derived stem cells

Normal human mesenchymal stem cells (MSCs) were obtained from the bone marrow (BMMSCs) of eight healthy orthopaedic patients undergoing hip replacement surgery. Approximately 10 ml of bone marrow were placed in culture plates, and the fibroblastic-like, plastic adherent fraction was isolated following multiple passages and was used in our experiments. The BMMSCs were further cultured and expanded in α-minimum essential medium (Gibco BRL, Invitrogen, Carlsbad, CA, United States), supplemented with 10% fetal calf serum (FCS; PromoCell, Heidelberg, Germany) and 2% penicillin/streptomycin mixture (Pen/Strep, 10,000 IU/ml; PromoCell), by incubation at 37°C in 5% CO_2_ atmosphere. Medium replacement was performed every third day and, upon reaching 80% to 90% confluence, the cells were passed using 0.25% trypsin-EDTA (ethylenediaminetetraacetic acid) solution (Sigma, St. Louis, MO, United States) followed by centrifugation (10 mins, 300 ×*g*) and were replated in T75 culture flasks at a density of 10000 cells/cm^2^ to ensure optimal proliferation.

#### 2.2.3 Characterization of human mesenchymal stem cells by flow cytometry

The expression of cell surface antigens of human ADSCs and BMMSCs (third passage) was assessed by flow cytometry. Briefly, preconfluent cells were detached from the culture dishes with trypsin-EDTA buffer and washed 2 times in PBS containing 1% FBS. Then, ∼10^6^ cells/ml were incubated for 30 min at 4°C with the appropriate monoclonal mouse anti-human fluorescein isothiocyanate (FITC)-conjugated antibodies to CD34, CD45, CD90, CD105 (Beckman Coulter, Germany), or phycoerythrin (PE)-conjugated antibodies to CD31, CD44, CD73 (BD Pharmingen, Franklin Lakes, NJ). Cells incubated with an identical concentration of FITC-, PE-conjugated mouse IgG isotype antibodies (BD Pharmingen) served as negative controls. A minimum of 10000 events were acquired on Gallios, Beckman Coulter ^®^ flow cytometer, and the results were analyzed using the Kaluza software (BD Biosciences).

#### 2.2.4 Tri-lineage differentiation of human mesenchymal stem cells

ADSCs and BMMSCs were characterized for their potential to differentiate into adipocytes, osteoblasts and chondroblasts, after being kept in culture for 3 weeks under specific supplemented cultured medium. Adipocytes, osteoblasts and chondroblasts were evidenced after differentiation induction by Oil Red O staining of lipid droplets, Alizarin Red staining of calcium deposits, and Alcian Blue staining of acid mucopolysaccharides, respectively.

### 2.3 Stem cell-derived extracellular vesicles

#### 2.3.1 Preparation of extracellular vesicles

After reaching 80% confluency, ADSCs and BMMSCs at passage five were rinsed with PBS and cultured in serum-free medium for 48 h. The conditioned medium was collected and centrifuged to isolate and purify extracellular vesicles (EVs). Briefly, the conditioned medium was centrifuged at 2500 g for 10 min s at 4°C to remove cellular debris, followed by a centrifugation at 16000 g for 5 min s at 4°C to remove apoptotic bodies. Then, the supernatant was collected and ultracentrifuged at 100000 g for 20 h at 4°C, and pelleted EVs (EV-ADSCs or EV-BMMSCs) were washed with PBS at 100000 g for 180 min s at 4°C and re-suspended in PBS. Finally, two different samples were performed, one with all EVs from ADSCs and the other with all EVs from BMMSCs. These were maintained at -80°C until further analysis. To establish the EV concentration, their protein concentration has been determined by BCA (bicinchoninic acid) method.

#### 2.3.2 Characterization of extracellular vesicles

Size distribution of EVs (EV-ADSCs or EV-BMMSCs), including microvesicles (100nm–1000 nm) and exosomes (50nm–100 nm), was measured by DLS using a Zetasizer Nano ZS ZEN3600 (Malvern Instruments, Malvern, United Kingdom) equipped with a solid-state He-Ne laser at 633 nm wavelength. All measurements were undertaken in triplicates at 25°C. Data processing and analysis were performed using Zetasizer software version 7.03.

The presence of the specific markers CD63^+^ (for exosomes) (ThermoFisher) and AnnexinV+ (for microvesicles) (SantaCruz Biotechnology, INC.) was evaluated by flow cytometry (Alexandru et al., 2020).

The structure and size of EVs (microvesicles + exosomes) isolated from the culture medium from both ADSCs and BMMSCs were investigated by transmission electron microscopy (TEM, FEI Talos F200C) utilizing negative staining technique according to the protocol described by Sorop et al. (2020). Briefly, isolated EVs (EV-ADSCs or EV-BMMSCs) were resuspended in 30 mM HEPES buffer, pH 7.4, containing 100mM KCl, and a 5 µL drop of this suspension was placed on a carbon coated copper grid (100 mesh; Agar Scientific), incubated for 2 min at room temperature (RT) and stained with 2% uranyl acetate. Image acquisition was done at RT using a 200kV Talos F200C TEM (ThermoFisher Scientific).

#### 2.3.3 MicroRNA profiling in extracellular vesicle samples using qPCR arrays

The total RNA was extracted from the human stem cell-derived EVs (EV-ADSCs or EV-BMMSCs) with miRNeasy Mini kit (Qiagen, Germany) according to the manufacturer’s instructions. The yield and purity of total RNA were determined using a spectrophotometer (NanoDrop 2000C, Thermo Fisher Scientific). A 260/280 OD ratio >1.8 was considered an indicator of acceptably pure RNA, relatively free of protein. Subsequently, the RNA samples (1 μg) were reverse transcribed to cDNA with the miScript II RT kit (Qiagen, German). The miScript PCR SyBR Green PCR kit (Qiagen) and miScript miRNA PCR array Human Cardiovascular Disease (MIRN-104Z) were used to run the Q-PCRs. Microarray results were normalized using six snoRNA/snRNA miScript controls (SN1/2//3/4/5/6). ΔCT value for each miRNA profiled in the plate was calculated using the formula ΔCT = C_T_^miRNA^–AVG C_T_^SN1/2/3/4/5/6^. ∆∆C_T_ for each miRNA across EV-ADSCs and EV-BMMSCs samples was calculated using the formula: ∆∆C_T_ = ∆C_T_ (EV-BMMSCs) – ∆C_T_ (EV-ADSCs). Fold-change for each miRNA from EV-ADSCs to EV-BMMSCs was calculated as 2^(−∆∆CT)^.

#### 2.3.4 Individual miRNA reverse-transcription and real-time quantitative PCR

RNAs from EVs isolated from conditioned media of human ADSCs and BMMSCs at passage five were diluted 4:1 with RNase-free water. To increase the amount of cDNA and improve the sensitivity of the TaqMan^®^ qPCR reaction, a preamplification step was performed. For this purpose, we made a preamplification primer pool targeting the same miRNAs, that were reverse transcribed, containing 5 μL of each individual 20×TaqMan^®^ Small RNA Assay diluted in Tris-EDTA. The preamplification reaction mix contained 3.75 μL of preamplification primer pool, 2.5 μL cDNA, 12.5 μL 1× TaqMan^®^ universal PCR MasterMix (2×), and 6.25 μL DEPC-treated water. Reverse-transcription was done with miRNA-specific stem-loop primers of miRNA TaqMan assays using TaqMan microRNA Reverse-Transcription Kit (Applied Biosystems, Thermo Fisher Scientific).

TaqMan miRNA assays (Applied Biosystems, Thermo Fisher Scientific) were used according to the manufacturer’s instructions to assess the levels of *Homo sapiens* hsa-miR-21-5p (ID 477975_mir), hsa-miR-30e-5p (ID002223), hsa-miR-93-5p (ID002139), hsa-miR-124-3p (ID000446), hsa-miR-126-3p (ID 002228), hsa-miR-143-3p (ID 002249), hsa-miR-146a-5p (ID000468), hsa-miR-155-5p (ID 002287), hsa-miR-181a-5p (ID000480), hsa-miR-181b-5p (ID001098), hsa-hsa-miR-185-5p (ID002104), hsa-miR-210-3p (ID000512), hsa-miR-221-3p (ID 000524), hsa-miR-222-3p (ID477982_mir), hsa-miR-223-3p (ID4427975_mir), and cel-miR-39-5p (ID000200) for normalization.

Real-time quantitative PCR was performed on ViiA7 real-time PCR system (Applied Biosystems, Life Technologies) using pre-amplified probes of miRNAs and miRNA Real-Time PCR Master Mix. For each sample, triplicate measurements were done on 96-well plates. The expression level of each individual miRNA was determined relative to cel-miR-39 and calculated using the 2^−ΔΔCq^ method (ΔΔCq = ΔCq^hsa-miRNA EV−BMMSCs^-ΔCq^hsa-miRNA EV−ADSCs^), then log-transformed for the statistical analysis.

### 2.4 Cardiomyocyte co-incubation with stem cell-derived extracellular vesicles in hypertrophic conditions

In order to investigate the effects of EV-ADSCs or EV-BMMSCs on cardiac hypertrophy, hiPSC-CMs were incubated with 200 nM AngII and 10 ng/ml TGF-β1 for 6 h, and then 100 μg/mL EV-ADSCs or EV-BMMSCs (Barile et al., 2014) were added in the culture along with these hypertrophic stimuli for additional 42 h.

#### 2.4.1 Extracellular vesicle uptake by recipient cardiomyocytes

EVs (EV-ADSCs or EV-BMMSCs) were labelled with red fluorescent membrane linker dye PKH26 (Invitrogen, Waltham, MA, United States) according to the manufacturer’s instructions. Briefly, 20 μL EV suspended in PBS were mixed with 1 ml of diluent C and 1 ml of PKH26 stock solution (4×10^−6^M/L) and incubated at 37°C for 5 mins, and the reaction was stopped by adding an equal volume of FBS. After incubation, EVs were washed with PBS via ultracentrifugation to remove unbound dye at 100000 g for 180 mins at 4°C. Finally, DAPI-stained cardiomyocytes (hiPSC-CMs) were incubated for 48 h with PKH26-labelled EVs and subsequently analysed under a Zeiss Axiovert microscope (Carl Zeiss, Jena, Germany) to determine the level of EV uptake by recipient cardiomyocytes (Georgescu et al., 2016).

### 2.5 Hypertrophic specific markers and inflammatory molecules in cardiomyocyte hypertrophy; role of extracellular vesicles

#### 2.5.1 Detection of cardiac hypertrophic biomarkers in cardiomyocytes by immunofluorescence staining

For fluorescence microscopy, Pluricytes Cardiomyocytes (hiPSC-CMs) were cultured for 7 days on glass coverslips in four well culture slide (Corning) coated with fibronectin (10 μg/ml in PBS containing Ca^2+^ and Mg^2+^) and then stimulated for 48 h with 200 nM AngII and 10 ng/ml TGF-β1 in the absence or presence of 100 μg/ml EV-ADSCs or EV-BMMSCs. Subsequently, both unstimulated and stimulated hiPSC-CMs were washed with PBS, fixed in 2% paraformaldehyde and permeabilized in buffer (2% BSA, 0.5% Triton-X-100 in PBS) for 20 min. Then, the cells were blocked in buffer (3% BSA in PBS) for 45 mins and incubated overnight at 4°C with primary antibodies diluted in blocking buffer. Next, the cells were washed 3 times in PBS, incubated with alexa fluor-conjugated secondary antibodies diluted at 1:1000 in blocking buffer for 60 min s at RT in dark, washed again 3 times (10 min each time) in PBS and counterstained with DAPI (Georgescu et al., 2012). The images were acquired using an Epifluorescence Microscope (Zeiss Axio Observer.Z1). The following antibodies were used: (Nadruz, 2015) primary antibodies: rabbit polyclonal anti-cardiac troponin I (cTnI) (ThermoFisher Scientific), mouse monoclonal anti-α-smooth muscle actin (α-SMA) (Cell Signalling Tehnology), rabbit polyclonal anti-atrial natriuretic peptide (ANP) (ThermoFisher Scientific), mouse monoclonal anti-Connexin 43 (Cx43) (ThermoFisher Scientific), mouse monoclonal anti-alpha-1 type I collagen (COL1A1) (Santa Cruz Biotehnology) and mouse monoclonal anti-macrophage migration inhibitory factor (MIF) (Santa Cruz Biotehnology); (Varagic and Frohlich, 2002) secondary antibodies: goat-anti rabbit IgG-FITC (Invitrogen) and donkey-anti mouse IgG-Alexa Fluor 567 (ThermoFisher Scientific).

#### 2.5.2 Evaluation of gene expression of molecules by Real-Time qPCR analysis

Total RNA from Pluricytes Cardiomyocytes (unstimulated hiPSC-CMs or hiPSC-CMs stimulated for 48 h with 200 nM AngII and 10 ng/ml TGF-β1 in the absence or presence of 100 μg/ml EV-ADSCs or EV-BMMSCs) was isolated with TRIZOL (Invitrogen, United States). Samples of 1 μg RNA were reverse transcribed using High Capacity cDNA Reverse Transcription kit (Applied Biosystems, Foster City, CA), according to the manufacturer’s instructions. Quantitative PCR reactions were carried out on a ViiA7 real-time PCR system (Applied Biosystems, United Kingdom) using 0.2 μg cDNA, the SYBR Select Master Mix (Applied Biosystems), and the primer pairs listed in the Table 1. Each sample was run in triplicate. The mRNA levels of tested genes were normalized to GAPDH gene, and the relative expression of each gene was calculated using the ddCt method.

**TABLE 1:** Primer sequences used for Real-Time qPCR.

Primer name	Forward	Reverse
cFOS	5′- CCT​AAC​CGC​CAC​GAT​GAT​GT	5′- TCT​GCG​GGT​GAG​TGG​TAG​TA
cJUN	5′- GGC​GAT​TCT​CTC​CAG​CTT​CC	5′- TCG​ACA​TGG​AGT​CCC​AGG​A
SMAD3	5′- TCT​CCA​AAC​CTA​TCC​CCG​AAT​C	5′- CTG​CGA​GGC​GTG​GAA​TGT​C
SMAD2	5′- TGT​GAA​GAG​ACT​GCT​GGG​ATG​G	5′- TTA​CAG​TTT​TGA​GTG​GTG​ATG​GCT
NF-kB subunit	5′- CCATGGACGAACTGTTCC	5′- TCT​TGG​TGG​TAT​CTG​TGC​TCC
COL1α1	5′- AAGCCGAATTCCTGGTCT	5′- TCC​AAC​GAG​ATC​GAG​ATC​C
Fibronectin	5′- CCGTGGGCAACTCTGTC	5′- TGCGGCAGTTGTCACAG
ACTA2	5′-GGC​AAG​TGA​TCA​CCA​TGG​GA	5′-GTG​GTT​TCA​TGG​ATG​CCA​GC
MMP9	5′-GTG​CGT​CTT​CCC​CTT​CAC​TTT​CCT	5′- GGA​ATG​ATC​TAA​GCC​CAG​CG
TNF	5′- CCT​CTC​TCT​AAT​CAG​CCC​TCT​G	5′- GAG​GAC​CTG​GGA​GTA​GAT​GAG
IL6	5′- CCT​GAA​CCT​TCC​AAA​GAT​GGC	5′- TTC​ACC​AGG​CAA​GTC​TCC​TCA
GAPDH	5′- ACC​ACA​GTC​CAT​GCC​ATC​AC	5′- TCC​ACC​ACC​CTG​TTG​CTG​TA

### 2.5.3 miRNA database analyses

To find the interaction between previously identified miRNAs in EV-ADSCs or EV-BMMSCs and target mRNAs the miRWalk Database was used.

miRWalk is an open-source platform providing an intuitive interface that generates predicted and validated miRNA-binding sites of known genes of human, mouse, rat, dog and cow (Sticht et al., 2018) and is available from http://mirwalk.umm.uni-heidelberg.de/. The miRWalk algorithm used miRDB database (http://www.mirdb.org/).

Subsequently, the Database for Annotation, Visualization and Integrated Discovery (DAVID) analysis (https://david. ncifcrf.gov/) was used for gene functional analysis based on Gene Ontology (GO) (Dennis et al., 2003). *p* < 0.05 and fold enrichment, higher than 2, were set as the cutoff criterion.

### 2.6 Statistical analysis

Depending on the type of experiment the results were expressed as the means ± SD or means ± SEM. Comparisons of a single variable in more than two groups were analyzed by one-way ANOVA followed by Bonferroni multiple comparison tests (GraphPad Prism). Statistical analysis was performed using the paired and unpaired *t*-test between two groups, using SPSS 12.0 software (SPSS, Inc., Chicago, IL, United States). Values of *p* < 0.05 were considered to indicate statistically significant differences.

## 3 Results

### 3.1 Developing and characterization of the *in vitro* model of hypertrophic cardiomyocytes

**FIGURE 1:**
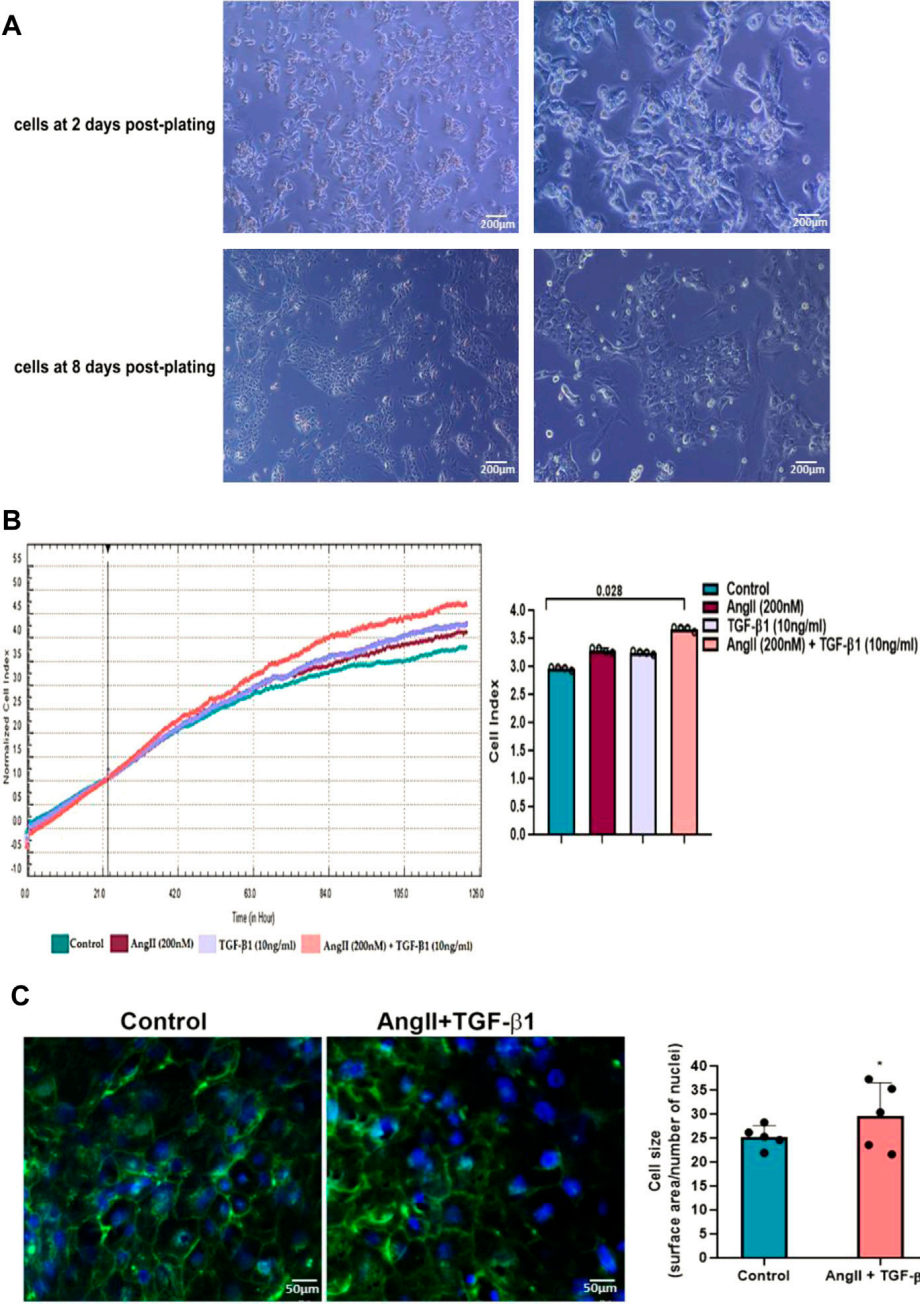
**(A)**. Morphology of hiPSC-derived cardiomyocytes produced *in vitro* in a 2D system. Representative phase contrast images after 2 and 8 days of culture of hiPSC-derived beating cardiomyocytes in monolayer in PC medium. The original magnification was ×10, respectively ×20 **(B)**. Real-time proliferation assay of unstimulated hiPSC-CMs (control cells) and hiPSC-CMs stimulated with AngII or TGF-β1 or with AngII + TGF-β1 for 48 hours using the xCELLigence® RTCA DP system. The impedance values were acquired automatically with the RTCA software and expressed as cell index values. Data are means ± SD of 4 independent experiments in duplicate for each experimental condition. The statistical significance, noticeably different, was represented as *P<0.05 value versus control cells. Two-way ANOVA test with Bonferroni post-test were applied **(C)**. Surface area quantification of unstimulated hiPSC-CMs (control cells) and stimulated hiPSC-CMs with AngII + TGF-β1 for 48 hours using FITC-conjugated wheat germ agglutinin (WGA) (green) to label cell membranes and DAPI (blue) to stain nuclei. The cell size was expressed as the ratio between the cell surface area and number of nuclei. Data are means ± SD of 5 independent experiments. Five different microscopic fields for each experimental condition were analysed. The statistical significance, noticeably different, was represented as *p<0.05 value versus control cells. T-test was applied.

To achieve a differentiated cardiomyocyte phenotype, hiPSC-CMs were cultured in PC medium optimized to promote cardiomyocyte structural and functional maturation 8 days after cultivation. Starting with the second day after cultivation the spontaneously beating phenotype of hiPSC-CMs was seen, and on the eighth day the mature phenotype of hiPSC-CMs, characterized by a more mature cell morphology and the formation of cardiac regulatory networks, was visualized under a phase-contrast microscope (Figure 1A).

Subsequently, to establish the model of hypertrophic cardiomyocytes, hiPSC-CMs were stimulated with either 200 nM AngII or 10ng/ml TGF-β1, or a combination of both (200 nM AngII with 10ng/ml TGF-β1) for 48 h, and hypertrophic growth of hiPSC-CMs was evaluated by real-time proliferation assay (Figure 1B).When hiPSC-CMs were stimulated with 200 nM AngII or with 10ng/ml TGF-β1 for 48 h, an increase in their impedance signals recorded by the xCELLigence^®^ RTCA DP system was observed over time, revealing a comparable induction of hypertrophic growth of cardiomyocytes, which was larger when hiPSC-CMs were incubated with a combination of both AngII and TGF-β1 (Figure 1B). The cell index values recorded after 48 h of incubation were significantly higher when hiPSC-CMs were stimulated with AngII in combination with TGF-β1 than in unstimulated hiPSC-CMs taken as control (*p < 0.05), and no differences were observed between the cell index values measured either for hiPSC-CMs stimulated with AngII or with TGF-β1 as compared to control cells (Figure 1B). As the Cell Index values of cardiomyocytes (hiPSC-CMs) increased significantly only in the presence of the two hypertrophic stimuli, added cumulatively to the culture medium, namely 200 nM AngII +10ng/ml TGF-β1, subsequent experiments were performed only for this experimental condition reported each time on the control sample (unstimulated hiPSC-CMs).

Importantly, since unstimulated and stimulated hiPSC-CMs do not proliferate in culture, (data supported by the MTT assay measurements and not shown), the hypertrophic growth of cardiomyocytes causes these significant different results between unstimulated hiPSC-CMs and stimulated hiPSC-CMs with AngII + TGF-β1 for 48 h. To confirm this, increasing the surface area of cardiomyocytes was evaluated by staining their membrane with FITC-WGA. As shown by fluorescence microscopy, the stimulation of hiPSC-CMs with the two hypertrophic agents, AngII and TGF-β1, caused a significant increase in the size of cardiomyocytes compared to control cells (unstimulated hiPSC-CMs) (*p < 0.05) (Figure 1C). Th*e* ratio between the WGA-stained cell surface area and number of DAPI-stained nuclei was used to define cell size, respectively cardiomyocyte hypertrophy (Figure 1C).

In order to characterize the *in vitro* model of hypertrophic cardiomyocytes, the following parameters were evaluated: signalling molecules relevant for the inducement of cardiomyocyte hypertrophy, intracellular reactive oxygen species (ROS) with role in cardiomyocyte hypertrophy and cardiac-specific biomarkers for cardiomyocyte hypertrophy.

**FIGURE 2:**
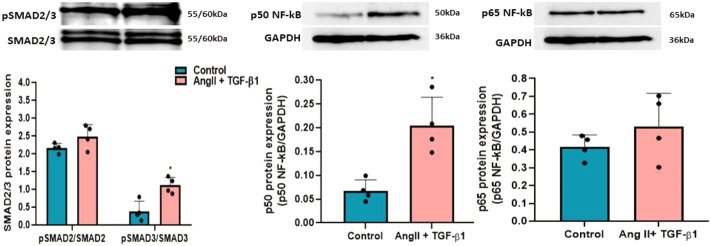
Western blot analysis for relative expression of pSMAD2/3 and p50/p65NF-kB molecules with role in hypertrophic growth of hiPSC-derived cardiomyocytes in culture. Histograms show a quantitative representation of the protein levels of pSMAD2/3 and p50/p65NF-kB obtained from hiPSC-CMs stimulated with AngII + TGF-β1 for 48 hours and from unstimulated hiPSC-CMs taken as control cells. Each value represents the mean ± SD. The statistical significance, noticeably different, was represented as *p<0.05 values versus control cells (*n* = 4). Statistical analysis was conducted using T-test. The grey intensity of related proteins was analysed by TotalLab TL120 program. The housekeeping β-actin protein was used as an internal control for protein normalization and monitor for equal loading.

To investigate whether Smad2/3 signalling pathway is activated in hiPSC-CMs stimulated with AngII + TGF-β1 for 48 h, the phosphorylation levels of Smad2 (Ser465/467) and Smad3 (Ser423/425) proteins were analysed by Western blot (Figure 2). The data showed that adding the two hypertrophic agents, AngII and TGF-β1, on hiPSC-CMs for 48 h increased the phosphorylation of Smad2 and Smad3 compared to unstimulated hiPSC-CMs, significant statistical differences obtaining only for pSmad3 (*p < 0.05) (Figure 2).

Also, the involvement of NF-kB transcription factor with the two subunits p50 and p65 in hypertrophic growth of cardiomyocytes was followed by Western blot analysis. The results revealed that p50NF-κB protein levels were significantly increased after stimulation of hiPSC-CMs with AngII + TGF-β1 for 48 h (*p < 0.05), while p65NF-κB protein levels had a slight, but insignificant growth in hiPSC-CMs stimulated with AngII and TGF-β1compared to those unstimulated (Figure 2). The representative Western blot original pictures for the expression of pSMAD2/3 and p50/p65NF-kB molecules can be found in Supplementary Figure S1.

**FIGURE 3:**
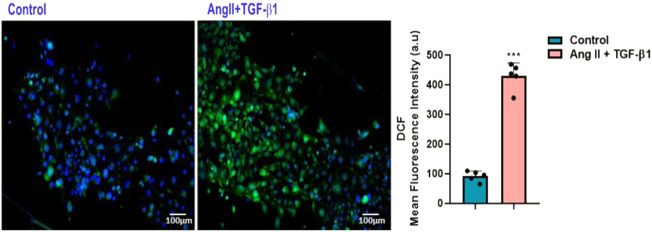
Measurement of DCF fluorescence as a measure of reactive oxygen species (ROS) in hiPSC-derived cardiomyocytes: (left side) Fluorescence microscopic images of intracellular ROS production by DCF staining (green) in hiPSC-CMs stimulated with AngII + TGF-β1 for 48 hours and unstimulated hiPSC-CMs taken as control cells; (right side) Average intensity of DCF fluorescence values in unstimulated and stimulated hiPSC-CMs. Nuclei were shown in blue fluorescence by DAPI dye staining. The images were quantified using the ImageJ program for 5 independent experiments. T- test was applied (***p<0.005 vs. control cells).

As for ROS generated by NADPH oxidase, it is known that they play a key role in Ang II–induced cardiac hypertrophy/remodelling (Bendall et al., 2002). To investigate whether ROS are involved in the hypertrophic effects of AngII + TGF-β1 on hiPSC-derived cardiomyocytes, we measured the intracellular ROS produced in these cells at 48 h after stimulation using DCFH-DA assay. The results showed that stimulation with both agonists increased cellular ROS production above the values obtained in control cells (unstimulated hiPSC-CMs), ***p < 0.005 (Figure 3).

**FIGURE 4:**
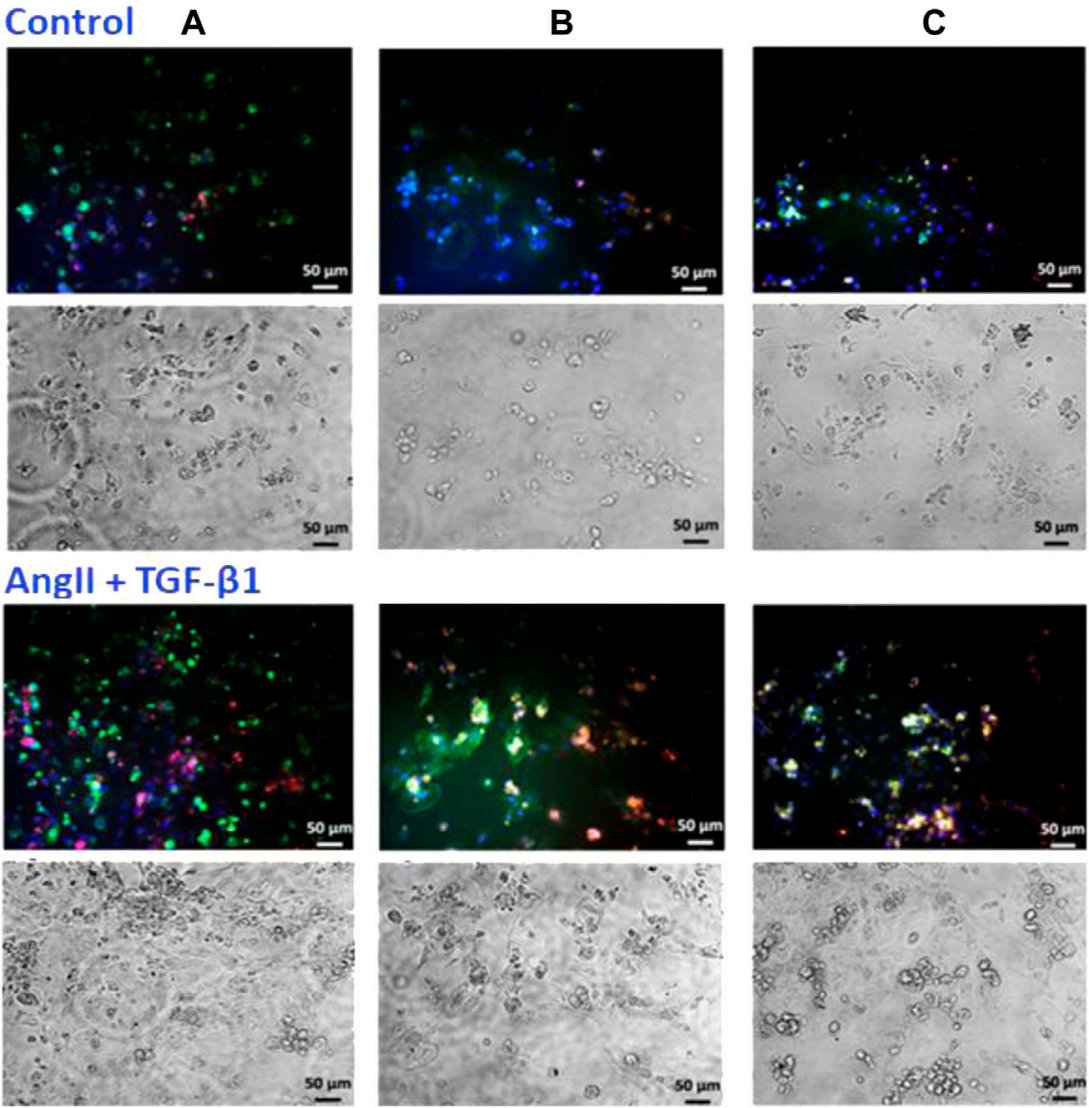
Immunofluorescence localization of **(A)** ANF (green) and MIF (red), **(B)** cTnI (green) and COL1A1 (red), and **(C)** α-SMA (green) and Cx43 (red) proteins in hiPSC-CMs incubated with 200 nMAngII + 10 ng/mLTGF-β1 for 48 hours and unstimulated hiPSC-CMs taken as control cells. Nuclei were stained with DAPI and shown in blue fluorescence. Five independent experiments were conducted in duplicate and five different microscopic fields for each tested molecule were analysed. The images were quantified using the ImageJ program.

To explore the cardiac biomarkers with role in cardiomyocyte hypertrophy, hiPSC-derived cardiomyocytes in culture were stimulated with AngII + TGF-β1 for 48 h, and the protein expression of atrial natriuretic factor (ANF), macrophage migration inhibitory factor (MIF), troponin I (cTnI), type I collagen (COL1A1), Connexin 43 (Cx43) and α-smooth muscle actin (α-SMA), were estimated by immunofluorescence performed on glass coverslips (Figure 4).

The analysis of fluorescence images and their quantification showed that protein expression for ANF, MIF, cTnI, COL1A1 and Cx43 were significantly higher in hiPSC-CMs stimulated with AngII and TGF-β1 then in unstimulated hiPSC-CMs taken as control cells, with ***p < 0.005 for ANF, MIF, cTnI, COL1A1 and **p < 0.01 for Cx43 (Figure 4 A, B, C).

Taken together, these results showed that when 200nMAngII were added together with 10ng/mLTGF-β1 on hiPSC-CMs for 48 h, cardiac hypertrophic markers ANF, MIF, cTnI, COL1A1, Cx43 and α-SMA have grown significantly (Figure 4).

### 3.2 The presence of stem cell specific markers in human ADSCs and BMMSCs

ADSCs and BMMSCs at the third passage were subjected to flow cytometry to determine whether the surface antigens specific to MSCs were present (Table 2).

**TABLE 2:** Flow cytometry analysis of ADSCs and BMMSCs lines confirmed the presence of MSC markers CD90, CD73, CD29, CD44, and CD105. Percentage of cells expressing the specific antigens for ADSCs and BMMSCs was analyzed using Kaluza software for 4 independent experiments.

Marker	Mesenchymal stem cells at third passage	
	ADSCs(%)	BMMSCs(%)
CD90	99.42 ± 3.68	83.86 ± 6.24
CD73	99.61 ± 6.77	82.06 ± 4.9
CD29	99.05 ± 1.63	84.95 ± 3.73
CD44	55.99 ± 20.51	69.72 ± 1.80
CD105	99.41 ± 6.28	79.07 ± 4.89

Flow cytometry analysis showed that human ADSCs exhibited positive reactions for CD73 (99%), CD90 (99%), CD105 (98%), CD44 (56%) and CD29 (99%), while BMMSCs displayed a relative lower expression of MSC markers, namely CD73 (82%), CD90 (84%), CD105 (79%), CD44 (70%) and CD29 (85%) (Table 2). Two flowcharts detailing the specific markers in human ADSCs and BMMSCs can be found in Supplementary Figures S2, S3. Together, these results revealed that ADSCs derived from lipoaspirate and BMMSCs from bone marrow aspirate used in our experiments were a homogenous population expressing stem cell specific markers.

#### 3.2.1 Mesenchymal stem cell differentiation analysis

**FIGURE 5:**
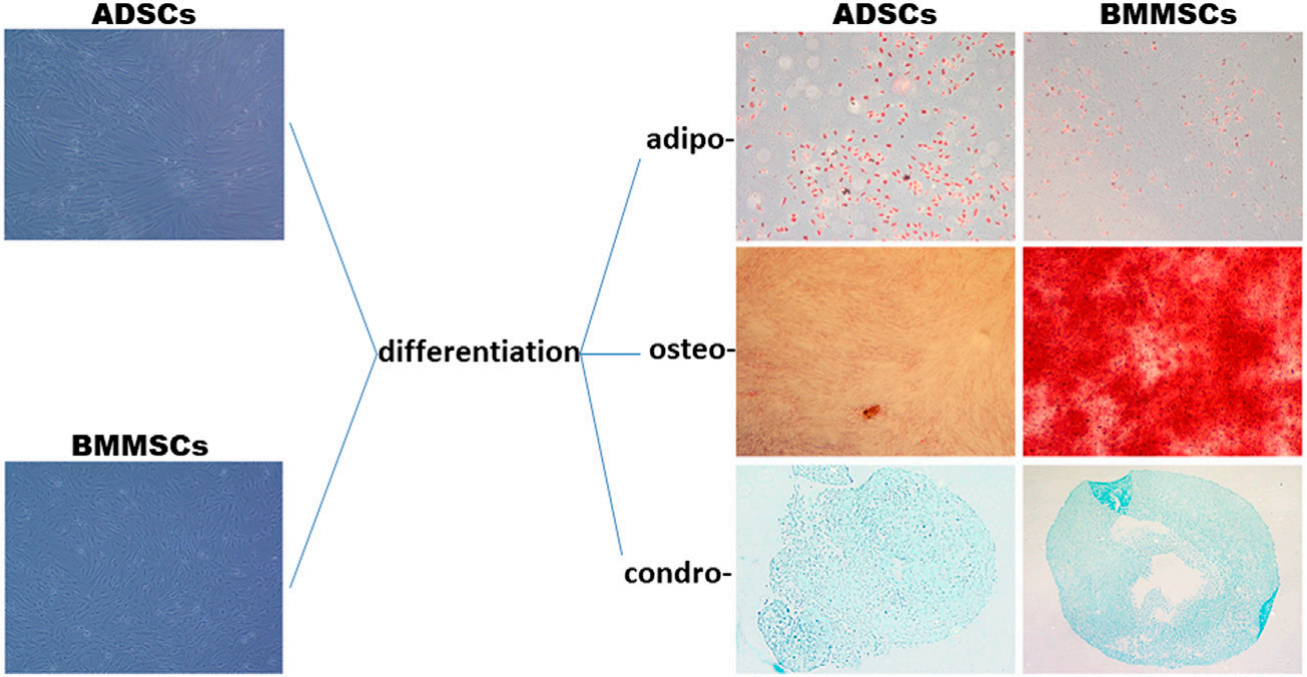
Stemness of ADSCs and BMMSCs was indicated by differentiation along three mesenchymal lineages, adipo-, osteo- and chondro-. Adipogenesis was evaluated by staining of lipid droplets with Oil Red O. Osteogenesis was evaluated by Alizarin Red S staining of the extracellular mineralized matrix. Chondrogenesis was determined by Alcian Blue staining of proteoglycan. All images were taken at ×20 magnification. Four independent experiments in duplicate were performed.

The stemness of human ADSCs and BMMSCS was confirmed by differentiation assays. There was an increased number of ADSCs that differentiated into mature adipocytes as can be seen from lipid-filled Oil Red O stained cells, and a significantly higher number of BMMSCs that differentiated into osteoblasts (Figure 5). There was no appreciable difference in cell morphology between ADSCs and BMMSCs that were incubated in specific media to differentiate into chondrocytes (Figure 5).

#### 3.2.2 Characterization of EVs isolated from conditioned medium of ADSCs and BMMSCs

To this purpose, EV-ADSCs and EV-BMMSCs, collected from conditioned medium of pre-confluent ADSCs and BMMSCs at passage five, were subjected to flow cytometry to determine whether the specific antigens characteristic for microvesicles and exosomes were present in the purified EV fraction.

**FIGURE 6:**
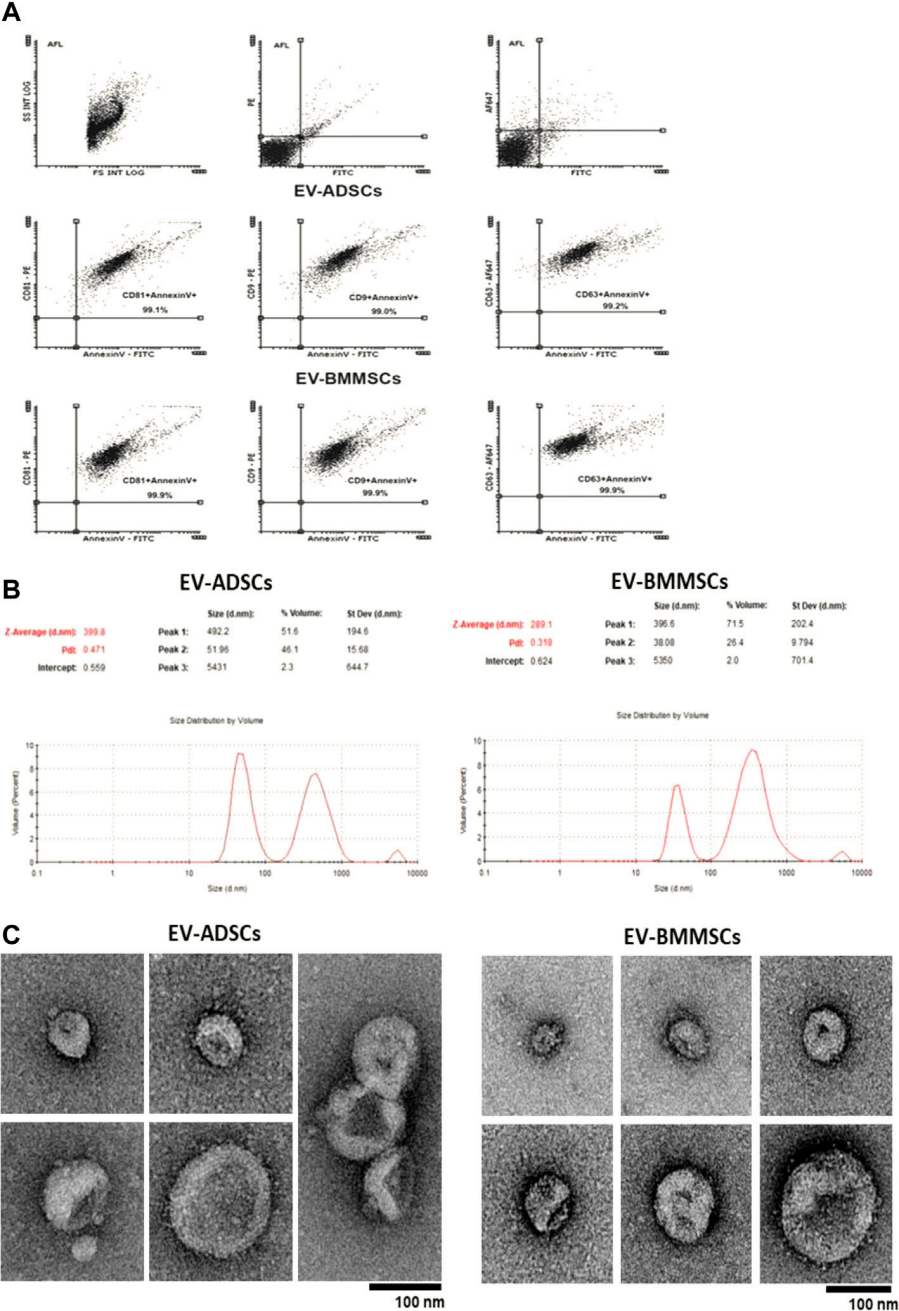
Detection and characterization EV-ADSCs and EV-BMMSCs: **(A)** Representative recordings obtained by flow cytometry highlights the presence of both exosomes (CD63^+^, CD9^+^, CD81^+^) and microvesicles (AnnexinV^+^) in the fractions of EV-ADSCs and EV-BMMSCs; **(B)** Representative records for particle size distribution of EV-ADSCs and EV-BMMSCs emphasizes two different populations represented by the two peaks, the first peak at the small size range (∼50–100 nm) specific to exosomes and the second at the high size range (∼100–1000 nm) specific to microvesicles (Y-axis represents the number of EVs and the X-axis represents the size of EVs); **(C)** Electron microscopy negative staining images of EV-ADSCs and EV-BMMSCs show round-shaped vesicles of different size in diameter. Scale bars reflect the magnification at the camera. Five independent experiments were conducted for each investigation.

Flow cytometry analysis revealed the presence of EVs with median size ≤1000nm, and of two different types of particles in EV fraction, namely microvesicles (AnnexinV+) and exosomes (CD9^+^, CD81^+^, CD63^+^) (Figure 6A). Double marking of EVs with AnnexinV, microvesicle specific marker, and with CD63, CD9, CD81, exosome specific markers could be explained by the formation of aggregates between microvesicles and exosomes. This phenomenon was also confirmed by the electron microscopy images taken on EV-ADSCs and EV-BMMSCs and shown in Figure 6C. Also, the electron microscopy negative staining images of EV-ADSCs or EV-BMMSCs showed a heterogeneous population of vesicles consisting of a range of sizes with singular or double membranes of differing densities (translucent light vs. translucent dark particles) (Figure 6B).

Subsequently, EV-ADSCs and EV-BMMSCs were characterized in terms of their size, using the Zetasizer Nano ZS device. Analysis on Zetasizer Nano ZS, showed that EV-ADSCs and EV-BMMSCs are represented by two populations of different sizes: exosomes (30–100 nm) and microvesicles (100–1000 nm) (Figure 6B). In EV-ADSCs, the population with an average size of ∼52 nm represented ∼46.1% of the total volume, while the population with an average value of ∼492 nm represented ∼51.6% of the total volume. In EV-BMMSCs, the population with an average size of ∼38 nm was ∼26.4% of the total volume, while the population with an average value of ∼396.6 nm was ∼71.5% of the total volume (Figure 6B). In conclusion, in EV-ADSCs there were an approximately equal number of microvesicles and exosomes, while in EV-BMMSCs the number of microvesicles was higher than that of exosomes. The difference in immunophenotypic character between ADSCs and BMMSC seen in Table 2 could be a justification for their different ability to release EVs comprising diverse proportions of small (exosomes) and large vesicles (microvesicles).

#### 3.2.3 Extracellular vesicle uptake by recipient hiPSC-derived cardiomyocytes in culture

**FIGURE 7:**
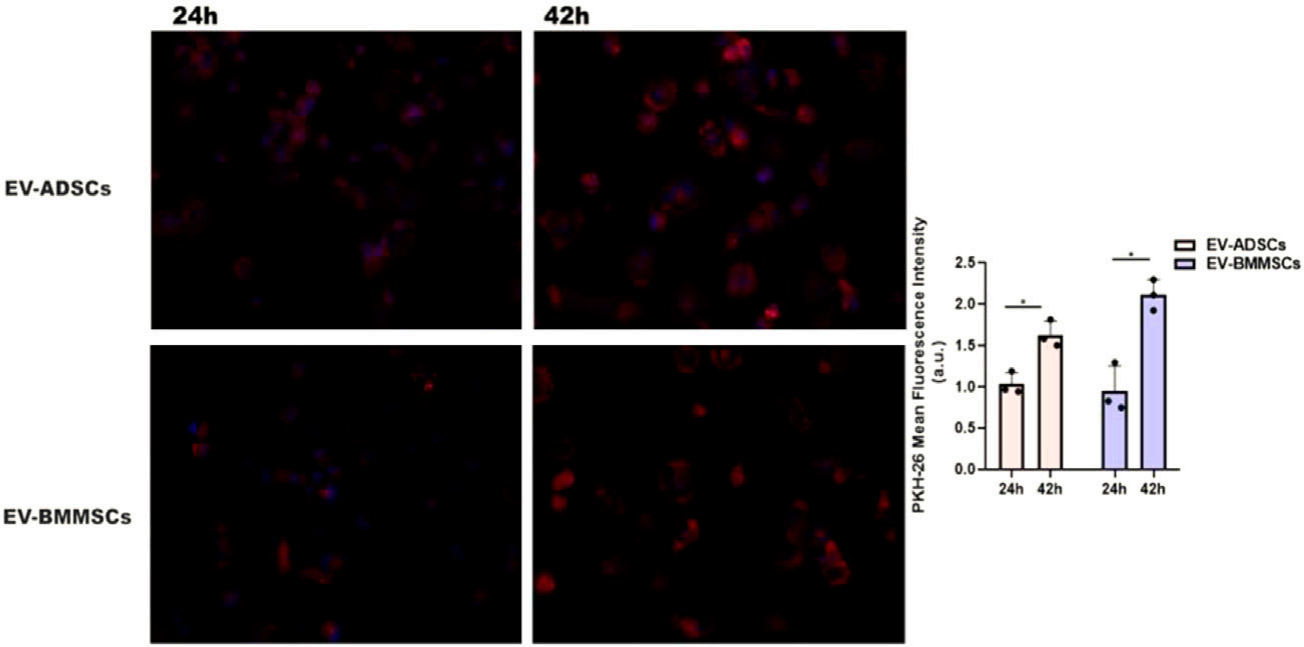
*In vitro* cellular internalization of EV-ADSCs or EV-BMMSCs from clinically healthy subjects by recipient hiPSC-derived cardiomyocytes. For these experiments, the DAPI-stained hiPSC-CMs were incubated with PKH26-labelled EV-ADSCs or EV-BMMSCs for 24 and 42 hours, respectively. Quantitative analysis of fluorescence intensity of PKH26-labeled EV-ADSCs or EV-BMMSCs revealed differential uptake capacity of the EVs at the two investigated incubation periods with elevated uptake observed at 42 hours, but without significant internalization differences for the two types of EVs, respectively EV-ADSCs or EV-BMMSCs. Three independent experiments in duplicate were conducted by fluorescence microscopy (the original magnification was ×20). Data shown as mean ± SD; One-way ANOVA (*p<0.05).

To demonstrate the incorporation of EVs by cardiomyocytes, hiPSC-CMs were incubated with either EV-ADSCs or EV-BMMSCs, obtained from clinically healthy subjects, labelled with PKH26, in the presence of hypertrophic stimuli AngII and TGF-β1 for 24 and 42 h, respectively. The presence of PKH26-labelled EV-ADSCs or EV-BMMSCs in hiPSC-CMs whose nuclei were stained with DAPI was investigated by fluorescence microscopy and analysed automatically using an ImageJ macro written for the task, yielding the ratio of total fluorescence/cell number. (Figure 9). The results showed that after 42 h of incubation, EV-ADSCs or EV-BMMSCs were taken up by cardiomyocytes in a significantly higher proportion (Figure 7). Moreover, fluorescence intensity analysis of PKH26-labeled EVs revealed that there are no significant differences in the internalization of the two types of EVs, respectively EV-ADSCs or EV-BMMSCs (Figure 7).

### 3.3 The effects of EV-ADSCs or EV-BMMSCs on cardiac biomarkers in hypertrophic cardiomyocytes

**FIGURE 8:**
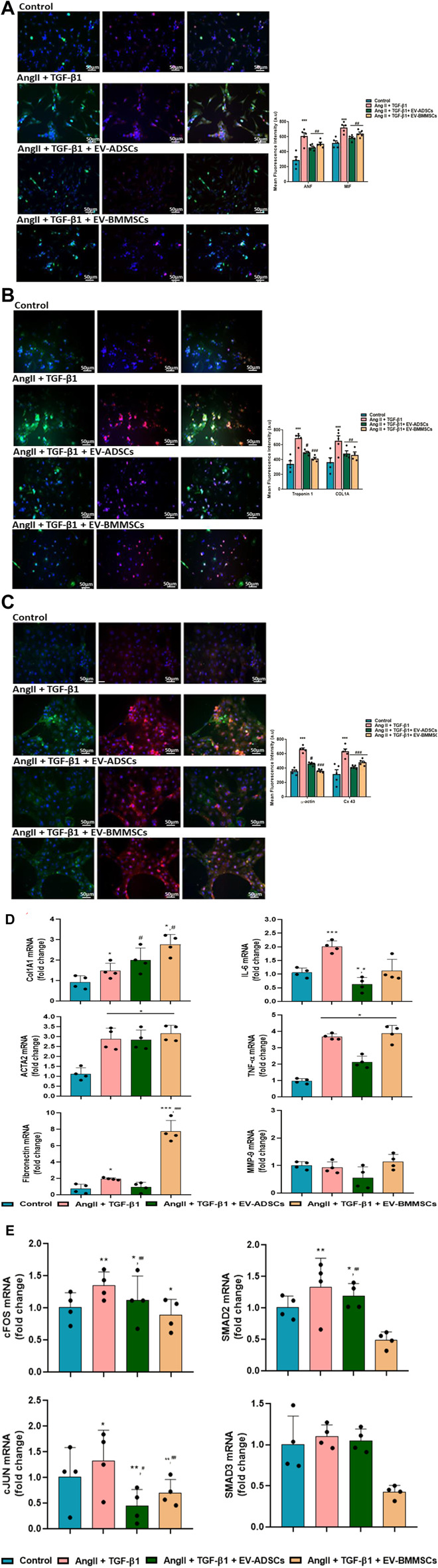
(Continued)

We were interested to investigate the role of internalized EVs in modulating the hypertrophic features in cardiomyocytes that have been stimulated with AngII and TGF-β1 for 48 h. For this purpose, hiPSC-CMs seeded on fibronectin coated cover slips, after 8 days when they established a contractile phenotype with spontaneously beating, were stimulated with AngII + TGF-β1 for 6 h, and then 100μg/mL EV-ADSCs or EV-BMMSCs were added in the culture along with these hypertrophic stimuli for additional 42 h. In parallel experiments, unstimulated hiPSC-CMs have been maintained in PC medium and were used as control cells. After 48 h unstimulated hiPSC-CMs and hiPSC-CMs stimulated as described above have been subjected to different protocols and molecules known to be involved in inflammatory and fibrotic processes associated with cardiac hypertrophy have been investigated. Notably, since in our study, the highest percentage of EV-ADSCs or EV-BMMSCs uptake by hiPSC-CMs was observed 42 h after incubation (Figure 8), we decided to evaluate the effects of EV-ADSCs or EV-BMMSCs on these key molecules only at this incubation period.

Firstly, hypertrophic specific markers, such as ANF, MIF, cTnI, COL1A1, α-SMA and Cx43, have been labelled with specific antibodies.

Immunofluorescence labelling of these cardiac-specific biomarkers in cardiomyocytes revealed that, the signal for hypertrophic specific marker ANF exhibited a 2.11-fold increase in hiPSC-CMs stimulated with AngII and TGF-β1 compared to control cells (unstimulated hiPSC-CMs) (*** *p* < 0.001) (Figure 8A). The ANF fluorescent signal have been observed to have a 0.25-fold decrease (^#^ *p* < 0.05), respectively 0.17-fold (^#^ *p* < 0.05), in hiPSC-CMs stimulated with AngII and TGF-β1 in the presence of EV-ADSCs, respectively EV-BMMSCs, as compared to ANF fluorescent level measured in hiPSC-CMs stimulated with AngII and TGF-β1 (Figure 8A).

Another hypertrophic marker MIF, an indicator of inflammatory process associated with hypertrophy in cardiac tissue, was noticed to be significantly enhanced 1.4-fold after stimulation of cardiomyocytes with AngII and TGF-β1 compared to unstimulated cardiomyocytes (***p < 0.005) (Figure 8A). EV-ADSCs or EV-BMMSCs added along with hypertrophic stimuli on hiPSC-CMs, decreased inflammatory milieu as can be seen from analysis of red fluorescence of MIF, i.e 0.19-fold (^#^ *p* < 0.05) in the case of EV-ADSCs and 0.11-fold (^#^ *p* < 0.05) in the case of EV-BMMSC versus the fluorescence levels measured in cardiomyocytes stimulated with AngII and TGF-β1 (Figure 8A).

As for the hypertrophic marker cTnI**,** its immunofluorescence images displayed that the hiPSC-derived unstimulated cardiomyocytes expressed low fluorescence levels, while cardiomyocytes stimulated with AngII and TGF-β1 exhibited a 2-fold increase in fluorescence levels (***p < 0.005) (Figure 8B). A considerable reduction of cTnI fluorescence levels was seen after incubation with EVs of hiPSC-CMs stimulated with AngII and TGF-β1 for 48 h, showing a 0.72-fold decrease (^#^ *p* < 0.05) in hiPSC-CMs incubated with EV-ADSCs and a 0.60-fold decrease (^###^ *p* < 0.005) in hiPSC-CMs incubated with EV-BMMSCs as compared to hiPSC-CMs that have been subjected only to hypertrophic stimulation (Figure 8B).

Further, COL1A1, a marker highly expressed in cardiac tissue being secreted by both fibroblasts and cardiomyocytes, was investigated in our *in vitro* experimental model (Figure 8B). The immunofluorescence results showed that hiPSC-derived cardiomyocytes exposed to AngII and TGF-β1 for 48 h presented an enhanced protein expression of COL1A1 1.8-fold higher than control cardiomyocytes (***p < 0.005) (Figure 8B). Adding either EV-ADSCs or EV-BMMSCs in parallel with hypertrophic stimuli on hiPSC-CMs in culture prevented the increase of COL1A1 protein expression showing a 0.3-fold decrease in both cases of EV incubation compared to cardiomyocytes exposed only to hypertrophic stimuli (^#^ *p* < 0.05) (Figure 8B).

In addition, α-SMA isoform known to be normally expressed in differentiating cardiomyocytes and a marker for myocardial hypertrophy in adult hearts (Kern et al., 2013) was examined in our experimental model of cardiac hypertrophy. Immunofluorescence analysis revealed a 1.46-fold increase in protein expression of α-SMA in cardiomyocytes stimulated with AngII and TGF-β1 compared to unstimulated cardiomyocytes (**p < 0.01) (Figure 8C). No significantly difference in terms of α-SMA staining were observed between hiPSC-derived cardiomyocytes incubated with EVs (irrespective of their origin, adipose tissue or bone-marrow) in the presence of AngII and TGF-β1 and cardiomyocytes that have been exposed only to hypertrophic stimuli (Figure 8C).

To explore the remodelling of gap junction during hypertrophy developed by hiPSC-derived cardiomyocytes in the presence of hypertrophic stimuli AngII and TGF-β1 for 48 h, the protein expression of Cx43 was followed by immunofluorescence (Figure 8C). The results showed that immune-detection of Cx43 in hiPSC-CMs stimulated with AngII + TGF-β1 was significantly increased 1.35-fold versus unstimulated cardiomyocytes taken as control (**p < 0.01) (Figure 8C). Notably, the fluorescence images displayed the presence of Cx43 in the perinuclear region in a higher concentration than at the boundary of the cells, and a substantially higher concentration in cardiomyocytes that manifest signs of hypertrophy. Interestingly, in the case of incubation with EV-BMMSC, the Cx43 fluorescence level remained unchanged from that observed in stimulated cardiomyocytes (Figure 8C).

Next, the gene expression of fibrotic markers COL1A1, ACTA2 and fibronectin was followed by qRT-PCR analyses.

COL1A1**,** whose protein expression levels were found to be elevated under the action of hypertrophic stimuli (as shown above in immunofluorescence images), also presented significant up-regulated gene expression levels under the action of the same stimuli, 1.58-fold higher than the unstimulated hiPSC-CMs taken as control cells, *p < 0.05 (Figure 8D). Interestingly, the presence of EV-ADSCs or EV-BMMSCs in the culture medium along with hypertrophic stimuli did not reduce the COL1A1 gene expression as it happened in the case of protein expression, but on the contrary both EV-ADSCs and EV-BMMSCs significantly enhanced the gene expression of COL1A1 compared to stimulated hiPSC-CMs, #*p* < 0.05 (Figure 8D).

The qRT-PCR analyses showed that hiPSC-CMs stimulated with AngII + TGF-β1 presented a significant upregulation of the gene expression of ACTA2 (actin alpha 2, smooth muscle**)** as compared to control cells (2.6-fold, *p < 0.05) (Figure 8D). EV-ADSCs or EV-BMMSCs added in the culture medium during the AngII and TGF-β1 treatment were not able to decrease the ACTA2 gene expression levels to the control condition (Figure 8D).

Fibronectin, an extracellular matrix protein that binds to integrin receptors and couples the cardiac myocytes to the basal lamina being involved in the cardiac remodelling process, was analysed. The results showed that the gene expression levels of fibronectin had a significant increase in stimulated cardiomyocytes compared to unstimulated ones (1.97-fold, *p < 0.05) (Figure 8D). The presence of EV-ADSCs did not generate any change in fibronectin gene expression measured in the presence of hypertrophic stimuli (Figure 8D). Surprising, the EV- BMMSCs induced a significant up-regulation of fibronectin gene expression even greater than that caused by hypertrophic stimuli (4- fold, ***p < 0.005) (Figure 8D).

Inflammatory markers IL-6, TNF-α and MMP-9 were also analysed in terms of change in the gene expression during *in vitro* cardiomyocyte hypertrophy inducement by AngII + TGF-β1, and the effects of 100μg/mL EV-ADSCs or EV-BMMSCs were questioned**.** Thus, it was found that the gene expression of IL-6**,** a pro-inflammatory molecule known to be involved in the cardiac hypertrophy, was significantly increased in hiPSC-CMs stimulated with AngII and TGF-β1 for 48 h compared to unstimulated cardiomyocytes in the control position (1.81-fold, *p < 0.05) (Figure 8D). Cardiomyocyte co-incubation with EV-ADSCs or EV-BMMSCs in hypertrophic conditions reduced IL-6 gene expression, but only EV-ADSCs induced a significant down-regulation of IL-6 inflammatory marker reported to AngII and TGF-β1 treatment (0.32-fold, ##*p* < 0.01) (Figure 8D). TNF-α also presented an up-regulated gene expression in hypertrophic cardiomyocytes versus control cardiomyocytes (3.67-fold, *p < 0.05) (Figure 8D), and the presence of EV-ADSCs or EV-BMMSCs in the culture medium together with hypertrophic stimuli failed to significantly reduce its expression (Figure 8D). Afterward the gene expression of MMP-9 was followed, and the results presented in Figure 8D showed that the transcript levels of MMP-9 were not different in cardiomyocytes stimulated with hypertrophic stimuli as compared with control cells, but these were significantly decreased in hiPSC-CMs incubated with EV-ADSCs in the presence of AngII and TGF-β1, #*p* < 0.05 (Figure 8D).

Overexpression of these investigated specific hypertrophic markers and fibrotic and inflammatory molecules 48 h after stimulation with AngII and TGF-β1 indicated that cardiomyocyte status in our *in vitro* experimental model advanced to pathological hypertrophy. The mechanism by which EV-ADSCs or EV-BMMSCs in the hiPSC-CM culture along with hypertrophic stimuli reduced all these key molecules was further investigated in our study by evaluating the effects of EV-ADSCs or EV-BMMSCs on transcription factors with role in cardiomyocyte hypertrophy.

Accordingly, the effects of EV-ADSCs or EV-BMMSCs on some nuclear transcription factors, such as SMAD2 and SMAD3 or cJUN and cFOS, with role in cardiomyocyte hypertrophic response induced by AngII and TGF-β1 were considered (Figure 8E).

Firstly, to evaluate the role of AngII signalling in cardiomyocyte hypertrophy, the mRNA levels of two key transcription factors cFOS and cJUN involved in gene regulation of fibrotic and inflammatory molecules associated hypertrophic response in cardiomyocytes were analyzed. The results showed that transcript levels of cFOS and cJUN were slightly increased in cardiomyocytes stimulated with Ang II and TGF-β1 compared to unstimulated cardiomyocytes considered as control test (1.26-fold, **p < 0.01 for cFOS and 1.33-fold, *p < 0.05 for cJUN) (Figure 8E). Also, the mRNA levels of cFOS were significantly reduced by EV-ADSC incubation (^#^ *p* < 0.05), while the mRNA levels of cJUN were significantly diminished by both EV-ADSCs (^#^ *p* < 0.05) and EV-BMMSCs (0.52-fold, *p* < 0.01) (Figure 8E), indicating that AP-1 transcriptional complex is essential for cardiomyocyte hypertrophy induced *in vitro* by Ang II or TGF-β1, but also for targeted therapy of cardiac hypertrophy.

Moving forward, we wondered whether SMAD2 and SMAD3, two molecules related to canonical signalling pathway induced by TGF-β1, were activated in hiPSC-CMs stimulated with Ang II and TGF-β1 for 48 h. The results showed that the gene expression of SMAD3 was not affected by hypertrophic stimuli, whereas the gene expression of SMAD2 had a significant increase compared to the control (1.3-fold, **p < 0.01) (Figure 8E). EV-BMMSCs proved to have beneficial effects on reducing SMAD2 (0.48-fold, ##*p* < 0.01) and SMAD3 (0.42-fold, ###*p* < 0.005) mRNA levels in cardiomyocytes stimulated with Ang II and TGF-β1 (Figure 8E).

Taken together, these results revealed that AP-1 activation is the molecular mechanism controlling gene regulation of markers associated with cardiomyocyte hypertrophy owed to Ang II or TGF-β1 rather than canonical signalling pathway dependent on transcriptional SMAD activation. EV-ADSCs seemed to interpose in a manner to reduce the transcript levels of cFOS and cJUN, whereas EV-BMMSCs were able to decrease activation of SMAD two and SMAD3 (Figure 8E).

Our *in vitro* study showed that the presence of EV-ADSCs or EV-BMMSCs in the hiPSC-CM culture along with hypertrophic stimuli, AngII and TGF-β1, reduced the protein expressions of hypertrophic specific markers, such as ANF, MIF, cTnI, COL1A1, and the gene expressions of IL-6 molecule involved in inflammatory process associated with cardiac hypertrophy and transcription factors SMAD2, SMAD3, cJUN, cFOS with role in cardiomyocyte hypertrophic response. It is important to note that, EV-ADSCs were more effective in reducing the protein expressions of hypertrophic and inflammatory markers, while EV-BMMSCs in reducing the gene expressions of transcription factors.

### 3.4 MicroRNA profiling in EVs from ADSCs and BMMSCs

Considering the results presented above which show the positive effects of EVs from ADSCs or BMMSCs, slightly different from each other, on the genes/proteins involved in inflammation and fibrosis processes associated with AngII and TGF-β1 treatment of hiPSC-derived cardiomyocytes, we wondered if these were the result of the miRNA content of EVs. Thus, we were interested to see if the therapeutic effect of EVs on hypertrophic cardiomyocytes is the result of the endogenous mechanism by which miRNAs from EV donor cells, ADSCs or BMMSCs, respectively, can change the translational profile of the recipient cardiomyocytes and modulate their morphology.

**FIGURE 9:**
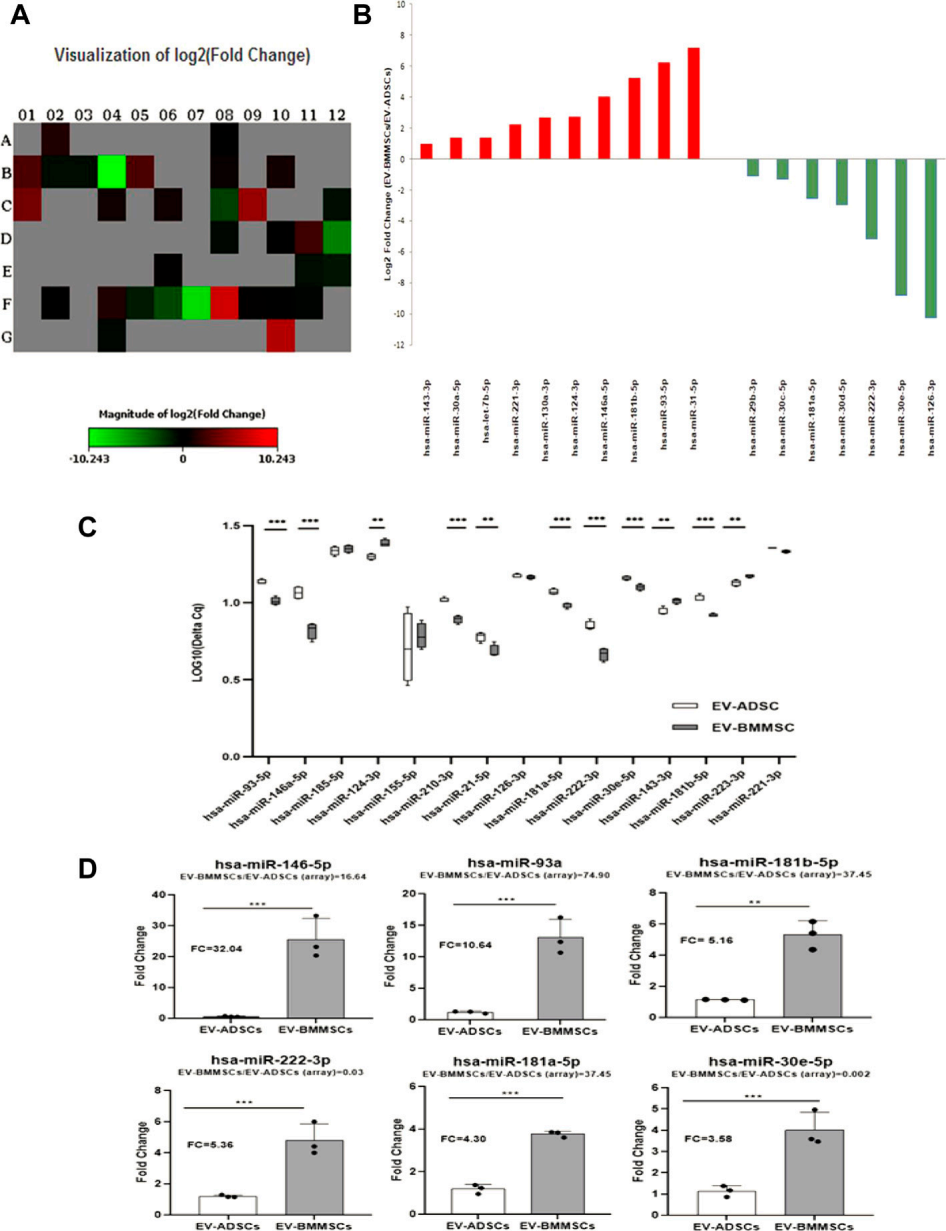
Differential expression profiling of miRNAs in EV-ADSCs and EV-BMMSCs. **(A,B)** miRNA profiling by miScipt miRNA PCR array: **(A)** Heat-map profile showing miRNA expression represented as log2 Fold Change, where Fold Change was calculated as normalized miRNA expression (2-ΔCq) in EV-BMMSCs divided to the normalized miRNA expression (2-ΔCq) in EV-ADSCs; **(B)** Bar plot representing log2 Fold Change of 17 differentially expressed miRNAs, of which 7 miRNAs were down-regulated (green) and 10 up-regulated (red) in EV-BMMSCs relative to EV-ADSCs; **(C,D)** TaqMan miRNA assays for selected miRNAs in EV-ADSCs and EV-BMMSCs: **(C)** Data for each selected miRNA are presented as boxplot of log-transformed values of Cq levels from four independent experiments; **(D)** Fold Change of miRNA expression in EV-BMMSCs relative to EV-ADSCs determined using TaqMan miRNA Assays, with bars represented as the mean ± standard error of the mean of three independent experiments. For comparison, fold change of EV-BMMSCs relative to EV-ADSCs found in miScript miRNA PCR array analyses was given for each miRNA when compared with EV-ADSCs, unpaired t-test analysis) (*p < 0.05, **p < 0.01, ***p < 0.005).

For this purpose, the screening of miRNA distribution in EV-ADSCs and EV-BMMSCs was performed by using Pathway-focused Human Cardiovascular Diseases miScript miRNA PCR array (Figure 9A). The differentially expressed miRNAs with *p*-value less than 0.05 and fold change higher than 2.0 or lower than 0.5 (threshold) were screened. In our analysis, among the 84 miRNAs on the array, only 34 miRNAs were present in both EV-ADSCs and EV-BMMSCs, as can be seen in heat-map profile of the PCR array (Figure 9A). However, from these commonly expressed miRNAs ten were significantly up-regulated and seven were significantly down-regulated in EV-BMMSCs compared to EV-ADSCs (Figure 9B). Eleven miRNAs on the array were detected only in EV-BMMSCs and the same numbers of miRNAs were uniquely expressed in EV-ADSCs.

Furthermore, the results obtained from miScript miRNA PCR array were validated by qRT-PCR using TaqMan miRNA Assays (Life Technologies). For this purpose, we selected hsa-miR-181a-5p, hsa-miR-185-5p, hsa-miR-21-5p from those miRNAs that were detected only in EV-ADSCs and also hsa-miR-143-3p, hsa-miR-146a-5p and hsa-miR-93-5p that were uniquely expressed in EV-BMMSCs. To this miRNA list were added those miRNAs which were expressed in both types of EVs, some stronger (Cq < 30) as hsa-miR-126-3p, hsa-miR-222-3p, hsa-miR-30e-5p and hsa-miR-181b-5p, and others weaker as hsa-miR-124-3p, hsa-miR-155-5p, hsa-miR-210-3p and hsa-miR-221-3p (Cq > 30). In order to enhance the amounts of detectable miRNAs, a pre-amplification step of cDNA obtained from probes of RNAs isolated from EV-ADSCs and EV-BMMSCs was performed.

The miRNA analysis thus selected, presented as boxplots in Figure 9C, revealed that the levels of most miRNAs were significantly higher in EV-BMMSCs compared to EV-ADSCs, and only hsa-miR-124-3p and hsa-miR-143-3p were significantly up-regulated in EV-ADSCs and EV-BMMSCs. However, no statistically significant differences were detected between the levels of hsa-miR-126-3p, hsa-miR-155-5p, hsa-miR-185-5p and hsa-miR-221-3p evaluated in EV-ADSCs and EV-BMMSCs.

When we compared the results obtained by miScript miRNA PCR array with data from qRT-PCR using TaqMan miRNA Assays for some miRNAs with the highest range of up-regulation in EV-BMMSC, specifically hsa-miR-146a-5p, hsa-miR-93-5p and hsa-miR-181b-5p, data were in a good agreement between the two analyses. Different results were observed for hsa-miR-222-3p, hsa-miR-181a-5p and hsa-miR-30e-5p that after analysis of miScript miRNA PCR array have been down-regulated in EV-BMMSCs compared to EV-ADSCs (FC = 0.03, FC = 0.17, FC = 0.002 respectively) (*p* < 0.005), meanwhile after individual assessment by TaqMan miRNA Assay they showed a significant up-regulation in EV-BMMSCs compared to EV-ADSCs (FC = 5.36, FC = 4.30, FC = 3.58 respectively) (*p* < 0.005) (Figure 9D). Separately, we analysed hsa-miR-124-3p and hsa-miR-143-3p who had FC = 6.57 and FC = 1.95 in screening assay, but after individual assessment by qRT-PCR had FC = 0.05 and FC = 0.34 (*p* < 0.01) in EV-BMMSCs versus EV-ADSCs. Another interesting finding was that hsa-miR-223-3p, a known miRNA enriched in MSC-derived exosomes, was not been detected by miSCRIPT miRNA PCR array and it was detected with enhanced expression in EV-ADSCs (*p* < 0.005).

#### 3.4.1 Functional enrichment analysis of the predicted target genes by specific miRNAs from EV-BMMSCs and EV-ADSCs

**FIGURE 10:**
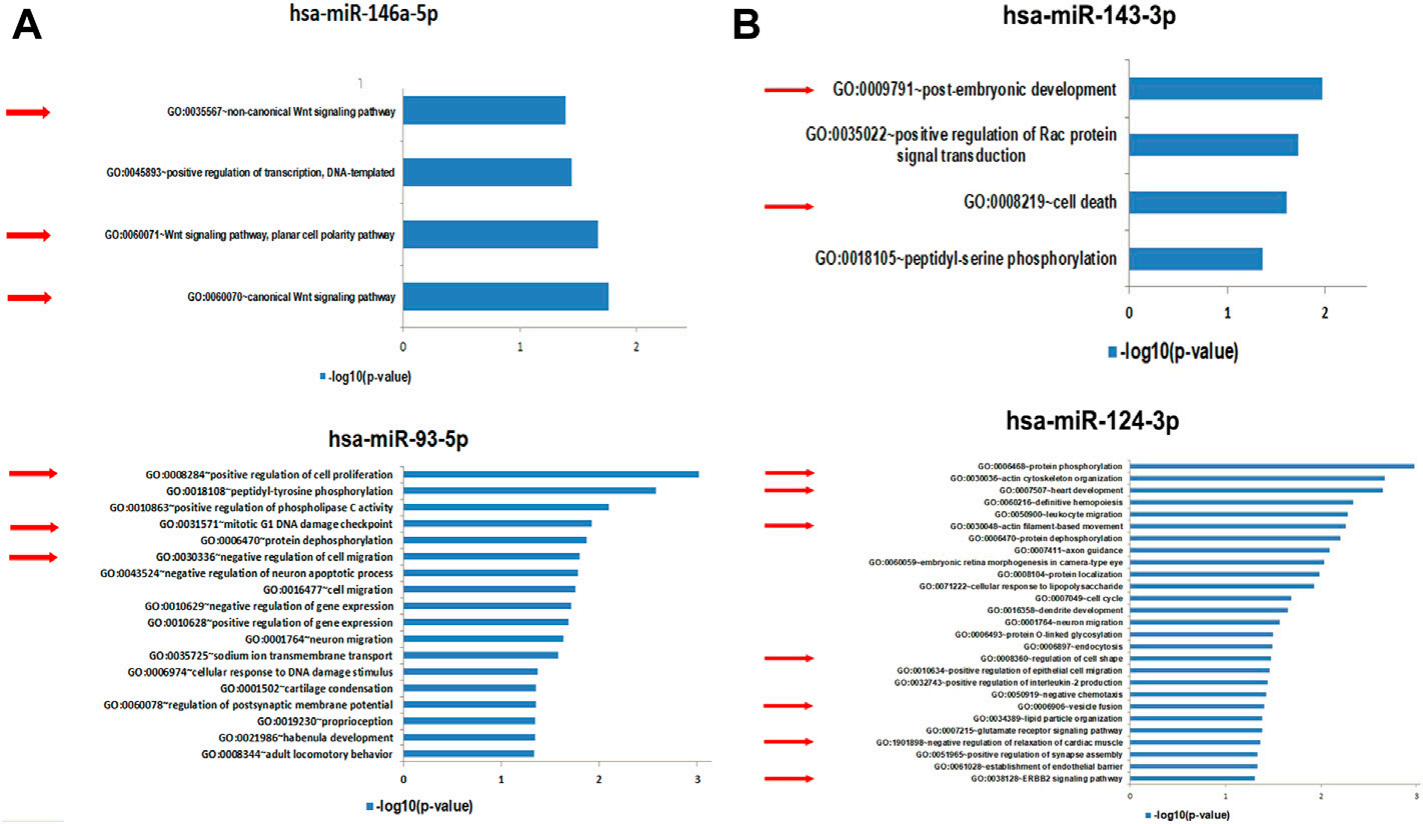
**(A)** Functional analysis for Gene Ontology (GO) biological processes associated with target genes identified by miRWalk algorithm to be potentially regulated by the selected microRNAs, miR-146a-5p and miR-93-5p, found to be up-regulated in EV-BMMSCs compared to EV-ADSCs. GO enrichment analysis of DEGs was retrieved using DAVID. The most significantly (*p* < 0.05) enriched GO terms in biological process are presented. All the statistically significant values were negative 10-base log transformed. Selected GO biological process terms are indicated by red arrows. DEGs, differentially expressed genes; GO, gene ontology **(B)**. Functional analysis for Gene Ontology (GO) biological processes associated with target genes identified by miRWalk algorithm to be potentially regulated by the selected microRNAs, miR-143-3p and miR-124-3p, found to be up-regulated in EV-ADSCs compared to EV-BMMSCs. GO enrichment analysis of DEGs was retrieved using DAVID. The most significantly (P<0.05) enriched GO terms in biological process are presented. All the statistically significant values were negative 10-base log transformed. Selected GO biological process terms are indicated by red arrows. DEGs, differentially expressed genes; GO, gene ontology.

After validation by qRT-PCR of miRNAs as being significantly up-regulated in EV-BMMSCs and EV-ADSCs, we were interested to identify the human genes that are potentially targeted by hsa-miR-93a-5p and hsa-miR-146a-5p found to be up-regulatied in EV-BMMSCs compared to EV-ADSCs (Figure 10A), and respectively by hsa-miR-143-3p and hsa-miR-124-3p found to be up-regulatied in EV-ADSCs compared to EV-BMMSCs (Figure 10B). The miRWalk algorithm that used published data base miRDB to predict the interaction between miRNA and mRNA was applied. Then we performed biological process enrichment analysis for the identified target genes of each miRNA using the Database for Annotation, Visualization, and Integrated Discovery (DAVID) platform, applying a double filtering for unadjusted *p*-values less than 0.05 and fold enrichment over 2.

The potential targets of hsa-miR-146a-5p, whose levels were increased in EV-BMMSCs compared to EV-ADSCs, may potentially regulate six biological processes, with most of them being associated with canonical and non-canonical Wnt signalling pathway (Figure 10A). Also, hsa-miR-93a-3p was found enriched in 19 biological processes including sodium ion transmembrane transport, regulation of gene expression, negative regulation of cell migration, mitotic G1 DNA damage checkpoint and positive regulation of cell proliferation (Figure 10A).

The targeted genes of hsa-miR-143-3p are associated with biological processes including the post-embryonic development, positive regulation of Rac protein signal transduction and cell death (Figure 10B).

Regulation of cell shape, negative regulation of relaxation of cardiac muscle, ERBB2 signalling pathway, endocytosis, cell cycle, actin filament-based movement, heart development and actin cytoskeleton organization were among the 28 pathways regulated by genes targeted by hsa-miR-124-3p (Figure 10B).

## 4 Discussion

Cardiovascular diseases are the leading cause of mortality and morbidity worldwide (Diseases and Injuries, 2020). Prolonged, untreated hypertension leads to myocardial hypertrophy that may progress to fibrotic remodelling and severe cardiomyocyte loss and finally to development of cardiac failure (Slivnick and Lampert, 2019). Because adult mammals do not possess the capacity for natural heart regeneration throughout their lifetime, therapies using mesenchymal stem cells (MSCs) has emerged in the last decade as a leading approach for a regenerative strategy to address cardiac disease (Karantalis and Hare, 2015). The beneficial effects of MSCs are due to their autocrine and paracrine activity (Sid-Otmane et al., 2020), or the released extracellular vesicles (EVs), the last gaining attention as potential new cell-free therapy tools in tissue repair (Raik et al., 2018; Gomzikova et al., 2019).

Based on these considerations, in the present study we asked whether EVs from subcutaneous adipose tissue stem cells (EV-ADSCs) or from bone marrow mesenchymal stem cells (EV-BMMSCs) have the capability to modulate the mechanisms involved in the cardiac hypertrophy.

Firstly, we developed and characterized an *in vitro* cellular model of hypertrophic cardiomyocytes that allowed the investigation of both the key mechanisms activated in cardiomyocytes hypertrophy and the therapeutic effects that stem cell-derived EVs could have on them. For this purpose, the hiPSC-CMs in culture were stimulated with AngII and TGF-β1 for 48h, in the absence or presence of 100μg/ml EVs.

Papers published recently evidenced that upregulation of Ang II stimulates TGF-β1 expression in the myocardium revealing the close interaction between Ang II and TGF-β mediated responses in the pathological cardiac hypertrophy (Schultz Jel et al., 2002; Rosenkranz, 2004; Bujak and Frangogiannis, 2007).

In our experiments, ventricular cardiomyocytes derived from hiPSC were cultured in PC medium and stimulated with 200 nM AngII, or 10ng/ml TGF-β1, or with 200 nM AngII and 10ng/ml TGF-β1 for 48 h in order to obtain the *in vitro* cellular model of hypertrophic cardiomyocytes. Following the evaluation of hypertrophic growth of hiPSC-derived cardiomyocytes through the (RTCA) xCELLigenceTM system and staining with WGA, we decided that the most hypertrophic inducer in our *in vitro* model was the combination of AngII (200 nM) with TGF-β1 (10ng/ml), and further experiments were performed in the presence of these two hypertrophic stimuli. In addition, we found that the NF-kβ and SMAD2 signalling pathways were activated in cardiomyocytes after stimulation with Ang II and TGF-β1. Moreover, our results revealed a significant increase in ROS levels and the overexpression of cardiac-specific biomarkers ANF, MIF, cTnI, COL1A1, Cx43, α-SMA after the exposure of cardiomyocytes to AngII and TGF-β1. All these results showed that hiPSC-derived cardiomyocytes exposed *in vitro* to AngII and TGF-β1 for 48 h manifest properties typically for cardiomyocytes from hypertrophied heart.

In the present study, using this *in vitro* cellular model of hypertrophic cardiomyocytes characterized above, we investigated the therapeutic potential of EVs from subcutaneous adipose tissue stem cells (EV-ADSCs) or from bone marrow mesenchymal stem cells (EV-BMMSCs) on the molecules involved in cardiac hypertrophy.

In a first stage, we wanted to characterize very well both ADSCs and BMMSCs, as well as EVs from them. The presence of stem cell specific markers (CD73, CD90, CD105, CD29 and CD44) in human ADSCs and BMMSCs grown in culture at passage three was proved by flow cytometry analysis. Also, stem cells from both sources presented high capacity to differentiate to adipose, osteo- and chondro-lineages under proper stimulation. Further, we demonstrated that EVs obtained by successive centrifuges from ADSCs and BMMSCs contain two populations of different sizes: small size vesicles entitled exosomes and medium size vesicles entitled microvesicles (Thery et al., 2018). The ratio of these vesicles in the secretome from ADSCs and BMMSCs was variable, with exosomes and microvesicles almost equal distributed in ADSCs, while in BMMSCs microvesicles represented a percentage of 71%. EVs from both stem cell sources exhibited the same characteristics: (Nadruz, 2015) they were positive for exosomes specific markers (CD9, CD81 and CD63) and also for Annexin V, a marker specific for microvesicles, that binds phosphatidylserine; (Varagic and Frohlich, 2002) they had a similar morphology with round shape with single or double membranes of differing densities (translucent light vs. translucent dark particles).

Using these valuable tools, hiPSC-CMs pre-treated with AngII and TGF-β1 for 6 h were incubated with 100μg/ml EV-ADSCs or EV-BMMSCs for additional 42 h to follow their effects on cardiomyocyte hypertrophy. Importantly, labelling of EVs with PKH26 enabled us to follow their entrapment inside the cardiomyocytes and no differences among EV-ADSCs or EV-BMMSCs regarding their intracellular abundance have been observed. Subsequently, the effects of EV-ADSCs or EV-BMMSCs on cardiac biomarkers in hypertrophic cardiomyocytes were explored. The obtained results showed that EV-ADSCs and EV-BMMSCs significantly decreased the protein expression of ANF, MIF, cTnI and COL1A1 and had no influence on α-SMA and Cx43, all these molecules being known as cardiac markers for cardiomyocyte hypertrophy that was induced by Ang II and TGF-β1. Next, the effects of EV-ADSCs and EV-BMMSCs on molecules with an important role in the fibrotic and inflammatory processes associated with cardiomyocyte hypertrophy were evaluated. Our data exhibited that the presence of EV-ADSCs or EV-BMMSCs in the hiPSC-CM culture along with Ang II and TGF-β1 diminished the gene expression of inflammatory molecules IL-6 and TNF-α and had no effect on gene expression of inflammatory molecule MMP-9 and fibrotic markers COL1A1, ACTA2 and fibronectin, whose levels of gene expression were significantly increased only in the presence of hypertrophic stimuli. Furthermore, in our study we followed the effects of EV-ADSCs or EV-BMMSCs on signalling molecules SMAD2 and SMAD3 or cJUN and cFOS with role in cardiomyocyte hypertrophy. In this sense we disclosed that the incubation of cardiomyocytes with EV-ADSCs or EV-BMMSCs reduced the mRNA levels of the transcription factors AP-1 (cJUN and cFOS), SMAD2 and SMAD3 found to be elevated under the action of hypertrophic stimuli. Also, from these experiments we concluded that the EV-ADSCs were more effective in reducing the protein expression of hypertrophic and inflammatory markers, while EV-BMMSCs in reducing the gene expression of transcription factors, and these effects could be attributed to their miRNA content capable to interact with target genes and thus to modulate biological processes relevant for cardiac function.

Referring to the existing studies in the literature on this topic, we found some papers that demonstrated that EV-BMMSCs have anti-fibrotic effects thus contributing to cardiac recovery (Chen et al., 2020; Wang et al., 2020). Also, in a paper we have recently published we showed that the treatment with EV-ADSCs or EV-BMMSCs alone or in combination with Smad2/3siRNA induced a significant decrease of expression of structural and inflammatory markers COL1A1, α-SMA, Cx43, VCAM-1 and MMP-2 at hypertensive-hyperlipidemic hamster that mimics human atherosclerosis (Ioana Karla Comarița et al., 2022). It was also shown in the same paper that transcription factors Smad2/3, ATF-2 and NF-kBp50/p65 involved in AngII and TGF-β1 signalling pathways are responsible for the changes in structural, functional and inflammatory markers of the vascular wall in the process of atherosclerosis (Ioana Karla Comarița et al., 2022).

In addition, different studies emphasized the importance of the AP-1 signalling pathway in cardiac function. In this sense it was shown that in neonatal cardiomyocytes deletion of each of the dimer components, cFOS or cJUN, depressed transcriptional activity of AP-1 (Omura et al., 2002; Jeong et al., 2005). Jeong et al., 2005 (Jeong et al., 2005) observed that when a dominant-negative form of cFOS was expressed, AP-1 activity in neonatal cardiomyocytes was stopped and this led to suppression of beta-myosin heavy chain and atrial/brain natriuretic peptides (ANP/BNP) expression. Related to the involvement of cJUN in cardiac hypertrophy, opposing data showed that cJUN-deficient hearts of mice subjected to pressure overload displayed pronounced fibrosis and increased myocyte apoptosis, suggesting that cJUN but not cFOS is required to induce a transcriptional program aimed at adapting heart growth upon increased workload (Windak et al., 2013). Together, these data support the notion that cJUN and cFOS, *via* activating AP-1, are necessary for induction of the pathological/fetal gene program and maintaining the hypertrophic phenotype of cardiomyocytes.

Therefore, stem cell-derived EV effects we observed on cJUN and cFOS, and thus on AP-1, could be responsible for decreasing mRNA levels of pro-inflammatory molecules IL-6, TNF-α in hypertrophic cardiomyocytes treated with EV-ADSCs or EV-BMMSCs, since AP-1 play a major role in the regulation of these molecules (Szabo-Fresnais et al., 2008). Analysis of the MIF gene sequence revealed also presence of AP-1 and NF-κB responsive elements (Baugh et al., 2002), meaning that these transcription factors are involved in the MIF expression that we found increased under the action of hypertrophic stimuli Ang II and TGF-β1 and decreased after EV-ADSC or EV-BMMSC treatment.

Taken together, the above results pointed that AP-1 transcriptional complex is essential for cardiomyocyte hypertrophy induced *in vitro* by Ang II and TGF-β1, but also may be a target for the stem cell-derived EV based therapy of cardiac hypertrophy.

Supporting our results on the positive effects of EV-ADSCs or EV-BMMSCs on inflammatory molecules, it was shown that microvesicles and exosomes present in the culture medium of ADSCs modulate chondrocyte metabolism to counteract the effects of IL-1β (Tofino-Vian et al., 2018).

Besides all these investigations, in an attempt to have a deeper understanding on the effects of EV-ADSCs and EV-BMMSCs on the molecules involved in cardiac hypertrophy we wondered if the miRNA content of EVs may contribute to differential effects observed on hiPSC-CMs incubated with AngII and TGF-β1. Thus, we examined the miRNA expression profile of EV-ADSCs and EV-BMMSCs. The results from screening revealed that 34 miRNAs were detected in EVs from ADSCs and BMMSCs, and from these ten were significantly up-regulated and seven were significantly down-regulated in EV-BMMSCs compared to EV-ADSCs. Besides, some miRs such as hsa-miR-126-3p, hsa-miR-222-3p, hsa-miR-30e-5p, hsa-miR-181b-5p, hsa-miR-124-3p, hsa-miR-155-5p, hsa-miR-210-3p hsa-miR-221-3p were expressed in both types of EVs and others only in EV-ADSCs (such as hsa-miR-181a-5p, hsa-miR-185-5p, hsa-miR-21-5p) or in EV-BMMSCs (such as hsa-miR-143-3p, hsa-miR-146a-5p, hsa-miR-93-5p). Further, those miRNAs displaying significant differences between EV-ADSCs and EV-BMMSCs in miScript miRNA PCR array were validated by qRT-PCR using TaqMan miRNA Assays.

As a result of this analysis, hsa-miR-146a-5p and hsa-miR-93-5p have been identified as being highly expressed in EV-BMMSCs as compared with EV-ADSCs, and hsa-miR-124-3p and hsa-miR-143-3p were significantly up-regulated in EV-ADSCs compared to EV-BMMSCs. The hsa-mir-146a-5p was the miRNA with the highest level in EV-BMMSCs and these results were confirmed by both techniques we used. A study by Song et al., 2017 (Song et al., 2017) found that human MSCs stimulated with Il-1β produced exosomes that transferred miR-146a to macrophages where it induced the down-regulation of M1 macrophage markers and up-regulation of M2 macrophage markers, suggesting a role of miR-146a in macrophage polarization. It was also shown that, exosomes containing miR-146a injected to miR-146a −/− knockout mice reduced TNF-α and Il-6 serum levels increased due to treatment with LPS, compared with miR-146a deficient exosomes injected to mice, demonstrating that exosomal miR-146a play an important role in moderating the inflammatory response (Alexander et al., 2015). Thus, hsa-mir146a-5p functions as an anti-inflammatory regulator in various immune cell types and the mechanism proposed is by repressing NF-κB and AP-1 signalling (Boldin et al., 2011). On the other hand, it was shown that overexpression of miRNA-146a in cardiomyocytes provoked cardiac hypertrophy and left ventricular dysfunction *in vivo*, whereas genetic knockdown or pharmacological blockade of microRNA-146a blunted the hypertrophic response and attenuated cardiac dysfunction *in vivo* (Heggermont et al., 2017).

As for the hsa-miR-93-5p we detected in EV- BMMSCs as being highly expressed, a very recent study has shown that suppression of hypoxia-induced autophagy and decrease of the inflammatory cytokine expression are the result of positive effects of miR-93-5p-containing exosomes isolated from ADSCs and injected to a mice model with myocardial infarction (Liu et al., 2021). These findings are in line with the report that EPC-derived EVs containing miR-93-5p have a beneficial effect by attenuating inflammation, vascular leakage and apoptosis in septic mice (He et al., 2020). Also, the beneficial effects observed for BMMSCs-exosomes containing miR-93-5p consist in stimulation of proliferation, migration and suppression of apoptosis in HaCaT cells damaged by H_2_O_2_ (Shen et al., 2022). Increasing evidence indicates that miR-93-5p may play an important role in the regulation of the cardiovascular system. However, the specific mechanism of miR-93-5p modulation in cardiomyocytes needs to be further clarified.

In addition, in connection with the hsa-miR-124-3p which we found to be highly expressed in EV-ADSCs, other studies have shown that overexpression of miR-124-3p alleviated increased ROS and decreased expression of p66Shc in doxorubicin-treated heart tissues and primary cardiomyocytes (Liu et al., 2019), and decreased TNF-α production in mouse macrophages (Sun et al., 2016). Interestingly, miR-124 can regulate the expression of Ikappa B (IκB) that was found to be necessary for the transactivation of a subset of NF-κB target genes (Yamazaki et al., 2008; Lindenblatt et al., 2009). On the contrary, high levels of miR-124-3p have been associated with increased expression of hypertrophic markers (cell size, ANP and BNP) in AngII-treated cardiomyocytes (Bao et al., 2017). When performed biological process enrichment analysis for the identified target genes of miR-124-3p using DAVID platform many targets of miR-124-3p have been identified. Regulation of cell shape, actin filament-based movement, heart development and actin cytoskeleton organization were also identified among the 28 pathways regulated by genes targeted by hsa-miR-124-3p.

Of interest in our miRNA profiling was the hsa-miR-143-3p that firstly was identified in higher concentration in EV-BMMSCs as compared to EV-ADSCs by screening assay, but when it was analysed individual by TaqMan its expression was significantly increased in EV-ADSCs. Our findings are in connexion with the papers that showed that human ADSCs express mir-143-3p that intervenes in the differentiation process of ADSC formation (Climent et al., 2015), and heart and vascular smooth muscle cells express miR-143/-145 gene cluster. Several clinical studies revealed that miR-143/-145 dysregulation is associated with many cardiovascular diseases, including essential hypertension, atherosclerosis, pulmonary arterial hypertension, and coronary artery disease (Caruso et al., 2012; Wei et al., 2013), and the regulation of the miR-143/-145 expression is controlled by TGF-β1/bone morphogen protein 4 (BMP4) network (Long and Miano, 2011; Climent et al., 2015). Investigations on heart morphogenesis in zebrafish demonstrated that miR-143 acts as a critical regulator of myocardial cell growth and elongation by directly targeting Adducin3 (Add3) protein which blocks actin filaments rearrangement (Deacon et al., 2010). Moreover, another study reported that miR-143 caused myocardial abnormalities in atrial and ventricular structure and defects in heart function (Miyasaka et al., 2011). Recently a functional screening study has probed 194 miRNAs differentially expressed in cardiac inflammatory disease for regulating cardiomyocyte size, cardiac fibroblast collagen content and macrophage polarization. Subsequent analysis of the results established that miR-145-3p can regulate the fibrotic response, whereas miR-223-3p, miR-486-3p, and miR-488-5p modulate macrophage activation and polarization in rat neonatal cardiomyocytes and cardiac fibroblasts exposed to phenylephrine to induce hypertrophy (Verjans et al., 2019).

*Limitations of the study.* Our results of the miRNA screening in EV-ADSCs and EV-BMMSCs gave us helpful information about the miRNAs they contain, which after release could interact with gene synthesis in the recipient cardiomyocytes. There are some inconsistencies in the fold changes of specific miRNAs between those calculated from miScript miRNA PCR array and those with TaqMan RT-qPCR. However, validation of results using complementary techniques remains technically challenging. Due to the low concentration of RNA that was isolated from the EVs, our miScript miRNA PCR array and TaqMan RT-qPCR analyses were performed on independent biological samples and this may in part account for the variability between sequencing and RT-qPCR results. Another inconvenient is that the primer technology is different for every of these techniques. Despite these limitations, it is notably that two independent groups of MSCs (ADSCs and BMMSCS from different patients) showed the inclusion of the same miRNA species, suggesting that these miRNAs reflect an accurate illustration of certain biological processes.

We hope that the results of future studies on EVs obtained by other methods from the same sources of MSCs will confirm at least some data already presented here on EVs obtained by ultracentrifugation. This is because EV study is a growing field but needs standardization for cell-free therapy applications in the future.

## 5 Conclusion

Combining the results from the *in vitro* experiments where the EVs from ADSCs and BMMSCs demonstrated their ability to reduce the expression of hypertrophic specific markers and molecules involved in inflammatory process associated with cardiac hypertrophy with the data from the bioinformatic analyses of miRNAs contained by EVs, we concluded that EV-ADSCs mainly promote cardiomyocyte remodelling processes, while EV-BMMSCs have a major effect on decreasing inflammation due to the delivery of the miRNAs they contain into the recipient cells.

The findings suggest that stem cell-derived EVs may be new promising tools for the treatment of cardiac hypertrophy known to be present in a number of heart diseases, including ischemic heart disease, hypertension, heart failure and valve diseases.

## Data Availability

The original contributions presented in the study are included in the article/Supplementary Material, further inquiries can be directed to the corresponding author.
