# Supplementary figures and images for: Stem cell‐derived extracellular vesicles reduce the expression of molecules involved in cardiac hypertrophy—In a model of human-induced pluripotent stem cell-derived cardiomyocytes
Source: Front Pharmacol. 2022 Oct 10;13:1003684. doi: 10.3389/fphar.2022.1003684 (PMC9589060; doi:10.3389/fphar.2022.1003684)

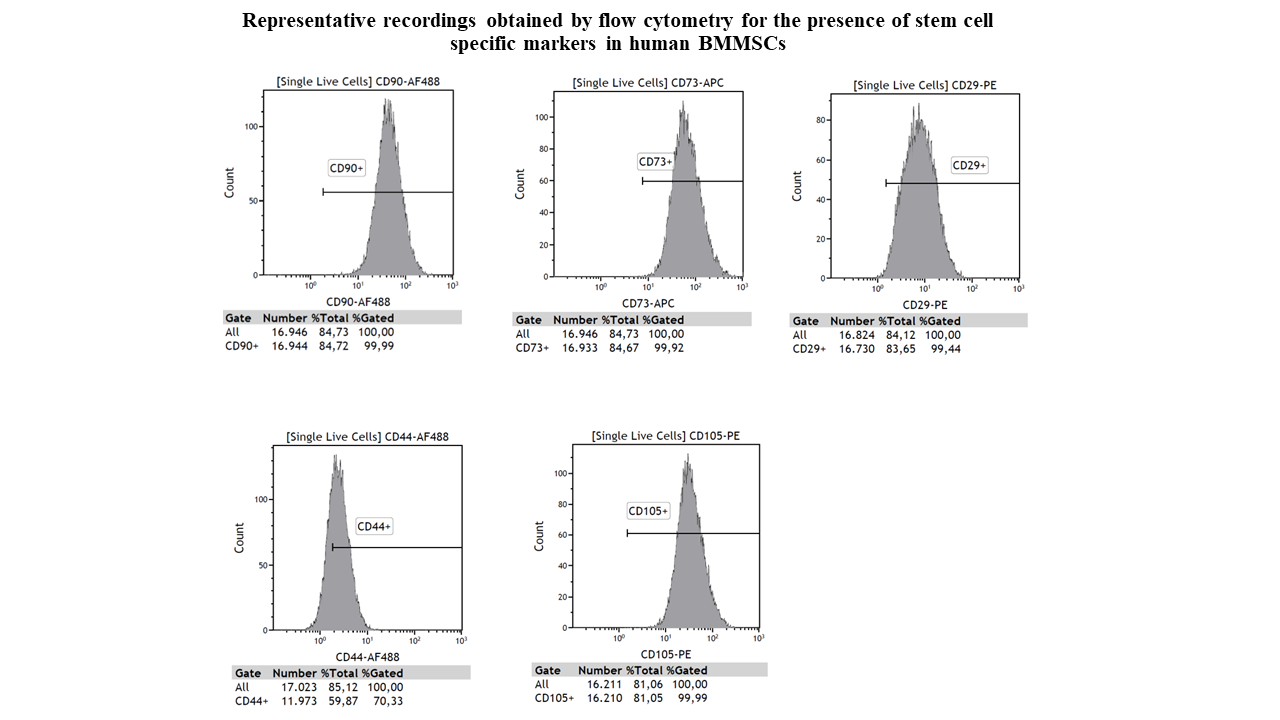

Supplement: Supplementary file 1 [file Image3.TIF]

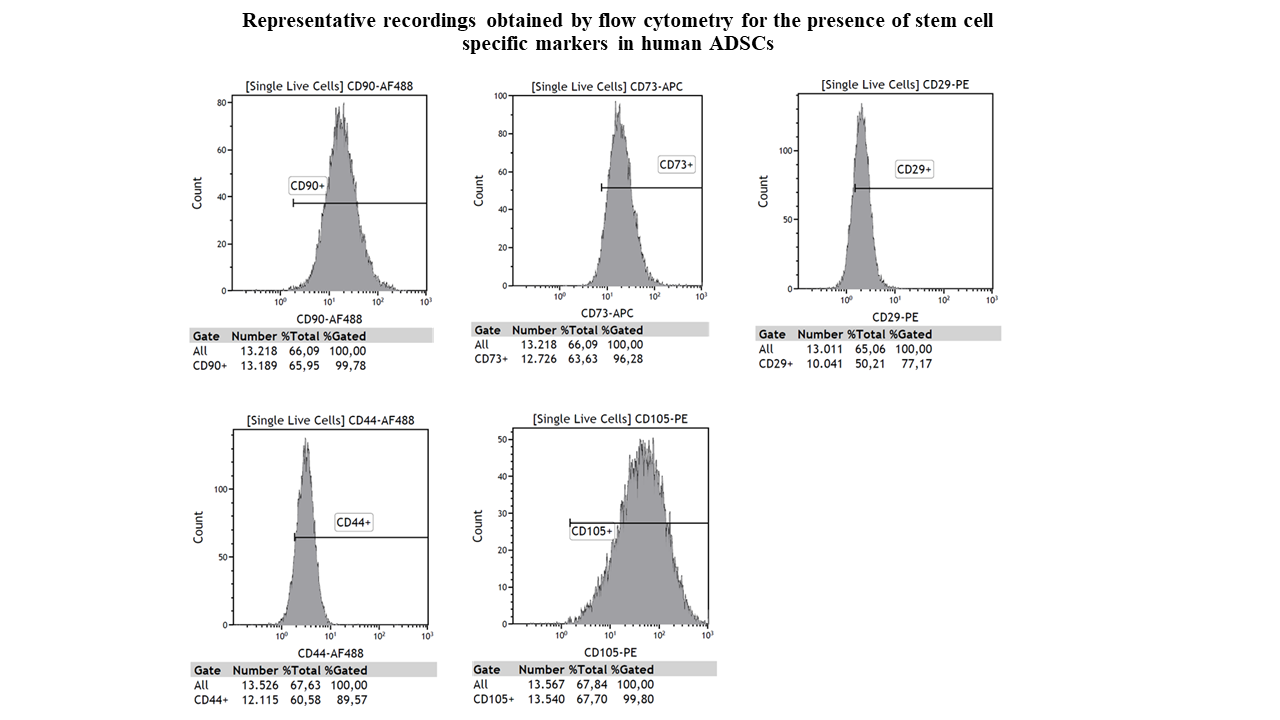

Supplement: Supplementary file 2 [file Image2.TIF]

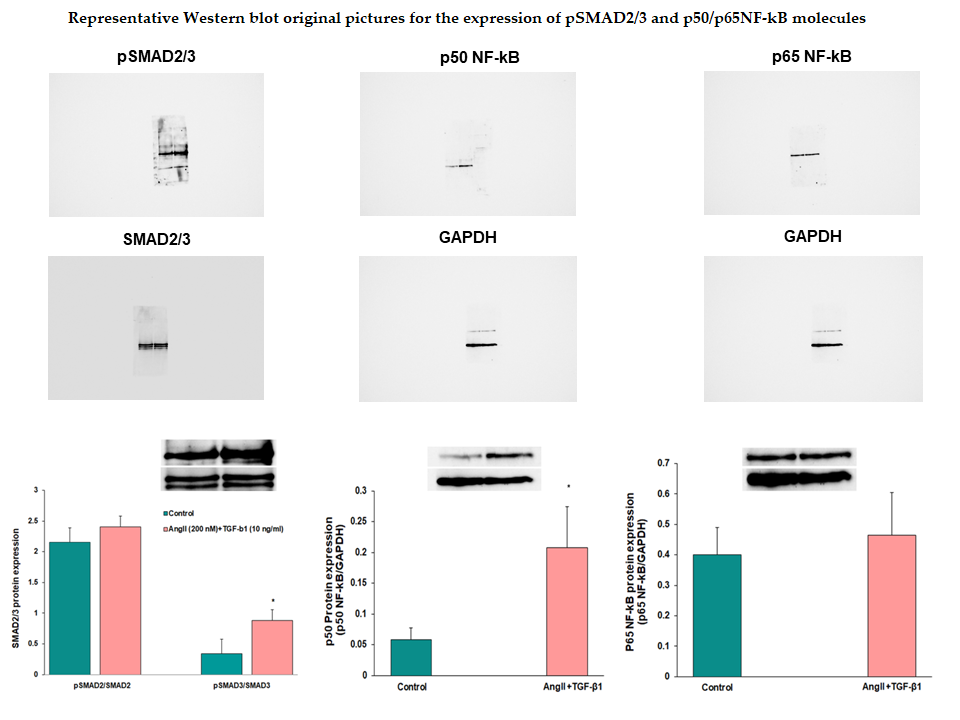

Supplement: Supplementary file 3 [file Image1.TIF]
